# Dietary Intake, Nutritional Adequacy and Food Sources of Total Fat and Fatty Acids, and Relationships with Personal and Family Factors in Spanish Children Aged One to <10 Years: Results of the EsNuPI Study †

**DOI:** 10.3390/nu12082467

**Published:** 2020-08-16

**Authors:** Casandra Madrigal, María José Soto-Méndez, Rosaura Leis, Ángela Hernández-Ruiz, Teresa Valero, Federico Lara Villoslada, Emilio Martínez de Victoria, José Manuel Moreno, Rosa M. Ortega, María Dolores Ruiz-López, Gregorio Varela-Moreiras, Ángel Gil

**Affiliations:** 1 Department of Nutrition and Food Science, Faculty of Pharmacy, University of Granada, Campus de Cartuja, s.n, 18071 Granada, Spain; casandram@correo.ugr.es (C.M.); mdruiz@ugr.es (M.D.R.-L.); 2 Iberoamerican Nutrition Foundation (FINUT), Av. del Conocimiento 12, 3 ª pta, Armilla, 18016 Granada, Spain; msoto@finut.org (M.J.S.-M.); ahernandez@finut.org (Á.H.-R.); agil@ugr.es (Á.G.); 3 Department of Pediatrics, Unit of Pediatric Gastroenterology, Hepatology and Nutrition University Clinical Hospital of Santiago, 15706 Santiago de Compostela, Spain; gvarela@ceu.es; 4 Instituto de Investigación Sanitaria de Santiago, IDIS, Santiago de Compostela, University Clinical Hospital of Santiago, 15706 Santiago de Compostela, Spain; 5 CIBEROBN (Physiopathology of Obesity and Nutrition), Instituto de Salud Carlos III (ISCIII), 28029 Madrid, Spain; 6 Spanish Nutrition Foundation (FEN), c/General Álvarez de Castro 20, 1ªpta, 28010 Madrid, Spain; tvalero@fen.org.es; 7 Instituto de Nutrición Puleva, Camino de Purchil 66, 18004 Granada, Spain; federico.lara@lactalis.es; 8 Department of Physiology, Faculty of Pharmacy, University of Granada, Campus de Cartuja, s.n, 18071 Granada, Spain; emiliom@ugr.es; 9 Institute of Nutrition and Food Technology “José Mataix,” Biomedical Research Center, University of Granada, Parque Tecnológico de la Salud, Avenida del Conocimiento s/n, Armilla, 18100 Granada, Spain; 10 Pediatric Department, University of Navarra Clinic, Calle Marquesado de Sta. Marta, 1, 28027 Madrid, Spain; jmorenov@unav.es; 11 Department of Nutrition and Food Science, Faculty of Pharmacy, Complutense University of Madrid, Plaza Ramón y Cajal s/n, 28040 Madrid, Spain; rortega@ucm.es; 12 Department of Pharmaceutical and Health Sciences, Faculty of Pharmacy, CEU San Pablo University, Urb. Montepríncipe, crta. Boadilla km. 5.3, Boadilla del Monte, 28668 Madrid, Spain; 13 Department of Biochemistry and Molecular Biology II University of Granada, University of Granada, Campus de Cartuja, s.n, 18071 Granada, Spain

**Keywords:** EsNuPI study, dietary fats, fats, lipids, essential fatty acids, food sources, pediatric nutrition, spanish children, dairy products, fortified milk

## Abstract

We aimed to determine the usual intake of total fat, fatty acids (FAs), and their main food sources in a representative cohort of the Spanish pediatric population aged 1 to <10 years (*n* = 707) who consumed all types of milk and an age-matched cohort who consumed adapted milk over the last year (including follow-on formula, toddler’s milk, growing-up milk, and fortified and enriched milks) (*n* = 741) who were participants in the EsNuPI study (in English, Nutritional Study in the Spanish Pediatric Population). Dietary intake, measured through two 24 h dietary recalls, was compared to the European Food Safety Authority (EFSA) and the Food and Agriculture Organization of the United Nations (UN-FAO) recommendations. Both cohorts showed a high intake of saturated fatty acids (SFAs), according to FAO recommendations, as there are no numerical recommendations for SFAs at EFSA. Also, low intake of essential fatty acids (EFAs; linoleic acid (LA) and α-linolenic acid (ALA)) and long-chain polyunsaturated fatty acids (LC-PUFA) of the n-3 series, mainly docosahexaenoic acid (DHA) were observed according to EFSA and FAO recommendations. The three main sources of total fat and different FAs were milk and dairy products, oils and fats, and meat and meat products. The consumption of adapted milk was one of the main factors associated with better adherence to the nutritional recommendations of total fat, SFAs, EFAs, PUFAs; and resulted as the main factor associated with better adherence to n-3 fatty acids intake recommendations. Knowledge of the dietary intake and food sources of total fat and FAs in children could help in designing and promoting effective and practical age-targeted guidelines to promote the consumption of EFA- and n-3 PUFA-rich foods in this stage of life.

## 1. Introduction

In recent years, changes in industrialization, urbanization, economic development, and market globalization have had a drastic impact on lifestyles and dietary patterns. Many populations are moving away from traditional to Westernized diets, characterized by the consumption of high-energy-density foods that are rich in fat, mainly of animal origin, and free sugars [1,2]. Consequently, there has been a significant increase in the prevalence of non-communicable chronic diseases (NCCDs), becoming the leading cause of mortality and disability worldwide [3,4,5].

Regarding fat intake, we know that Spanish children and adolescents consume high quantities of total fat when compared to current recommendations (34.6–38.9% of the energy intake (EI)) [4,6,7,8]. Large long-term cohort studies with follow-up and randomized clinical trials are providing strong evidence of the impact of fatty acids on health and conclude with several statements about the mechanisms by which some of them may have beneficial or adverse effects [9,10,11] Fats are precursors of biological molecules with critical metabolic functions; they are involved in the transport of fat-soluble vitamins and contribute to the integrity of cell membranes and tissue formation [12,13,14].

In childhood, fats are not only a prime source of energy, but also provide essential fatty acids (EFAs) and polyunsaturated fatty acids (PUFAs), which are critical for appropriate growth, cognitive development, physical activity, and prevention of NCCDs [12,15,16,17]. PUFAs of the n-3 and n-6 series have a protective effect on health, which goes beyond the mere cardiovascular effect. The brain, retina and other neural tissues are particularly rich in PUFAs; therefore, a sufficient intake is crucial for brain and neurological development, photoreception, reproductive system in the children [18,19]. During adulthood, the intake of PUFAs continues to be crucial, due to the relationship between n-3 PUFAs and several chronic diseases, cardiovascular, and inflammatory diseases [12,14,18,19]. High or deficient intake of fat can have repercussions on health in the short, medium, and long term [13,20,21].

Academic institutions, scientific societies, and national and international organizations, such as the World Health Organization (WHO), the Food and Agriculture Organization of the United Nations (UN-FAO), and the European Food Safety Authority (EFSA), have elaborated consensus documents with guidelines and recommendations for the consumption of fat and different FAs and their health effects [12,22,23]. In this sense, they warn that the consumption of fat should not exceed 35% of total energy. Regarding the intake of saturated fatty acids (SFAs), the EFSA and the Institute of Medicine (IoM) indicate as a nutritional objective that the contribution of SFAs should be as low as possible, without setting a maximum intake value [23,24]. The total n-6 PUFA recommendation is 5% to 10% of daily energy, and for n-3 PUFA, the recommendations vary among organizations [12,13,25]. For EFAs, the recommendation for linoleic acid (LA) is 4%, and α-linolenic acid (ALA) is 0.5% of total energy for children [26]. Although humans and animals have the ability to convert ALA to eicosapentaenoic acid (EPA) and docosahexaenoic acid (DHA), the efficiency of conversion is low, in particular to DHA; hence, high levels of EPA and DHA in blood or other cells are reached only when they are provided as such in the diet from the consumption of fish and fish oils, which are rich sources of these FAs [12,13].

In Spain, the National Dietary Survey on the Child and Adolescent Population (ENALIA study, 2012–2014) of young people 4 to 17 years old showed high fat intake, 34.6% of the total caloric value (TCV) of the diet, with a significant contribution of SFAs to the detriment of monounsaturated fatty acids (MUFAs) [4]. In the Anthropometry, Intake and Energy Balance Study (ANIBES), carried out with 213 participants 9 to 13 years of age, total fat and SFAs intake was much higher than recommended (38.9% and 13.1%, respectively) and, notably, there was a high contribution of MUFAs (16.0%) coming mainly from olive oil [7]. However, in the ALSALMA (“Alimentando la Salud del Mañana”) study of 1701 children 0–3 years of age, high consumption of protein and carbohydrates and low total fat was observed [27]. European data, such as the Identification and Prevention of Dietary- and Lifestyle-Induced Health Effects in Children and Infants (IDEFICS study) of children 2 to 9 years of age from 8 countries, show that the Mediterranean dietary pattern is inversely associated with childhood obesity [6]. Also, in the National Health and Nutrition Examination Survey (NHANES study) 2009–2014, a cross-sectional study of non-breastfeeding American children under 5 years of age, the intake of SFAs was higher than the recommendation (12.8% of TCV) [8]. It should be noted that in NHANES III, the presence of children at home was associated with higher consumption of total fat and SFAs [28]^.^

Despite the previous data, in Spain, there is still scarce information about current fat and fatty acid intake during childhood, as well as the main food sources of fat, especially in children under 5 years old.

In recent years, a lot of controversy about the contribution of milk fat to the intake of fat and SFAs have arisen [29,30]. Dairy is the main foodstuff in many countries, and it seems to be an essential source of calories and SFAs. Recent studies on the consumption of whole milk have shown an inverse association with weight increase and the risk of obesity and cardiovascular diseases (CVD) [31,32]. Other reviews support the importance of regular dairy consumption on linear growth and bone mineralization in children, given its rich nutrient profile, and in the prevention of some NCCDs later in life [25,33,34]. Besides the consumption of toddler and young children milk formulas, enriched and fortified milk within the Spanish pediatric population is increasing, and there is a lack of evidence whether the consumption of this type of milks is making any difference in nutrient intakes and if they are helping to reach the nutrient recommendations.

For all these reasons, it is interesting to find out whether dairy consumption is associated with a healthier dietary pattern from the first years of life and with a different contribution of fat and FAs to the diet, as well as its possible effects on growth. Also, it is of interest to find out whether the consumption of conventional milk and dairy products or adapted milk formulas and fortified dairy products has an impact on the intake of EFAs, PUFAs, and on the whole dietary profile. This will allow dietary recommendations for healthier patterns in children to be established, which will last into adulthood and have positive repercussions on health in the short, medium, and long term [35]. Based on that background, the EsNuPI study (in English, Nutritional Study in the Spanish Pediatric Population) was designed to determine the food consumption, nutrient intake, and physical activity and sedentary behaviors of Spanish children from 1 to <10 years old in urban areas with >50,000 inhabitants, distributed across nine geographical areas [36].

In particular, the aims of the present study are: (1) to determine the usual total fat and FAs intake in a representative cohort of the Spanish pediatric population in three age groups (group 1, 1 to <3 years old; group 2, 3 to <6 years old; and group 3, 6 to <10 years old) who consumed all types of milk and in an age-matched cohort of adapted milk consumers (including follow-on formula, toddler’s milk, growing-up milk, and fortified and enriched milks); (2) to evaluate to what extent these population groups meet the EFSA and UN-FAO recommendations for fat and FAs intake; (3) to ascertain the primary food sources providing fat and different families of FAs; and (4) to evaluate the influences of several determinants, i.e., family and personal factors, anthropometry, physical activity level (PAL), parental education, and socioeconomic status, on fat and FAs intake.

## 2. Materials and Methods

### 2.1. Study Design and Sample

The present study is based on data from the EsNuPI study. The complete design, protocol, and methodology of the EsNuPI study have been described in detail elsewhere [36].

Briefly, the EsNuPI study was a prospective, cross-sectional, observational study of a population cohort of children aimed at evaluating the dietary and nutrient intake and dietary patterns as well as physical activity and sedentary behaviors of Spanish children living in urban areas, distributed in 9 regions according to Nielsen Spanish areas.

Two cohorts with a total of 1514 children were selected for the EsNuPI study. The first cohort consisted of 742 children of the urban non-vegan Spanish population aged 1 to <10 years old consuming all types of milk in the last 12 months, and that was randomly selected (Spanish Reference Cohort, SRS). The second cohort of 772 children came from a “booster cohort” of urban non-vegan children aged 1 to <10 years old who regularly consumed “adapted milk” over the last 12 months (Adapted Milk Consumers Cohort, AMS) (including follow-on formula, toddler’s milk, growing-up milk, and fortified and enriched milks).

This research was conducted according to the guidelines of the Declaration of Helsinki and was approved by the University of Granada ethical committee (No. 659/CEIH/2018) and registered at ClinicalTrials.gov (unique protocol ID: FF01/2019). Written informed consent was obtained from all parents or caregivers before participation.

### 2.2. Dietary Data Collection

The dietary data collection was conducted in line with the EFSA European Union (EU) Menu Project guidance recommendations. This guidance document facilitates the collection of harmonized food consumption data from all EU member states [37].

Dietary information was collected through two days of food recall (one face-to-face and one telephone 24 h dietary recall (24 h DR)), including weekdays and weekend days, filled out by the parents or caregivers as a proxy to determine children’s dietary intake.

Parents or caregivers were instructed to recall all beverages and foods that children consumed on the day before the interview (at home, at school, and elsewhere outside the home), including details of the amounts, ingredients, brands, recipes, and preparation methods. The interviewer completed the 24 h DR with the parents using the “Tables of common home measures and habitual portion sizes for the Spanish population” [38] and a photoguide of common portion sizes of Spanish foods [39], built using the pilot study for the assessment of nutrient intake and food consumption among kids in Europe (PANCAKE) standards [40].

The dietary information was checked for clarity and completeness by the study nutritionists. Food, beverages, total energy, and nutrient intake were calculated from the collected food data using VD-FEN 2.1 software [41], which is based mainly on the Spanish food composition table [38]. To determine the milk and dairy product consumption a detailed extension of the composition of the different infant and adult milk types that currently exist in the market (260 items) was created, obtained from the official manufacturer websites, and added to the software VD-FEN 2.1 for the analysis of the energy and nutrients that these dairy products provide.

We classified all recorded food items according to the following 18 food groups: “milk and dairy products”, “cereals”, “meat and meat products”, “oils and fats”, “bakery and pastry”, “fruits”, “vegetables”, “sugars and sweets”, “ready to cook/eat”, “other dairy products”, “beverages”, “legumes”, “eggs”, “fish and shellfish”, “appetizers”, “cereal-based baby foods and supplements”, “nuts”, and “sauces and condiments”.

An analysis of the total amount of nutrients intake provided by all foods eaten by the population was performed. The population proportion method, according to Krebs-Smith, 1989 [42], was used to determine the contribution of each nutrient from the different food categories.

The analysis for the present study included daily intake of total fat, SFAs, MUFAs, PUFAs, omega-3 (n-3), omega-6 (n-6), and selected FAs: myristic acid (14:0), palmitic acid (16:0), stearic acid (18:0), palmitoleic acid (16:1 n-7), oleic acid (18:1 n-9), LA (18:2 n-6), alpha-linolenic acid (ALA) (18:3 n-3), arachidonic acid (AA) (20:4 n-6), EPA (20:5 n-3), docosapentaenoic acid (DPA) (22:5 n-3), and DHA (22:6 n-3).

### 2.3. Sociodemographic and Anthropometric Data

In the first stage of the study, the interviewers asked about sociodemographic data of the children and their parents or caregivers, including date of birth, gender, parental education (according to the International Standards Classification of Education), place of residence (Nielsen area), family income (€), parental occupation (according to National Classification of Occupations of Spain), lifestyle, physical activity, and sedentary behaviors. Additional questions were asked to confirm that the children met the inclusion criteria and their correct inclusion in the designed cohorts (SRS, AMS).

Weight and height measurements were reported by the parents or caregivers according to their most recently available pediatric health card. The anthropometric data were evaluated using the WHO international growth patterns [14]. WHO Anthro and WHO Anthro PLUS software (version 3.2.2, January 2011) were used to calculate the children’s z-scores (z-score Body Mass Index (BMI)/age and z-score height/age).

### 2.4. Physical Activity and Sedentary Behavior Habits

The questionnaire used for the EsNuPI study was modified and adapted from a questionnaire previously validated in children <10 years in Colombia [43].

Physical activity and sedentary behavior were reported, indicating all activities performed by the child per day for the last 7 days (one week), including hours of sleep and screen time, and were reported separately for weekdays and weekends. For more details, see the methodology of the EsNuPI study [36].

### 2.5. Evaluation of Plausible, Under-, and Over-Reporting (Misreporting)

In the present study, the EFSA protocol was used to assess misreporting [44], which is based on the work of Goldberg and Black [45,46]. This method evaluates the reported energy intake (EIrep) against the energy requirements for each child. EIrep is expressed as a multiple of the mean basal metabolic rate estimate (BMRest; calculated from Schöfield equations) and is compared with the energy expenditure of the studied population. The ratio EIrep: BMRest is referred to as the PAL. The assessment of misreporting was performed at both the cohorts and individual level, according to EFSA recommendations.

Subjects were identified as plausible, under-, or over-reporters of energy intake based on the ratio of EIrep to estimated energy requirement. According to these criteria, children with energy intake (EI) below the cutoff were considered as under-reporters, those with EI between cutoffs were considered plausible reporters, and those with EI above the cutoffs were considered high energy reporters or over-reporters. The detailed methodology of the EsNuPI study has been explained in previous publications [36,47].

In the present research, potential misreporting was not excluded from the database because the exclusion of misreporters did not result in differences in nutrient intake, so it did not significantly modify the results and conclusions of this study [44]. Nevertheless, the results of plausible reporters are presented later in the present document.

### 2.6. Statistical Analysis

Once the dietary information was collected, the reported 746 food items were grouped into 18 food groups and transformed into energy and nutrients for analysis.

Due to the collection of the two 24 h DRs, the variance of usual cohort intake is inflated by day-to-day variation in individual intake [48]. To eliminate the intra-individual variability of the data and obtain an estimate of the usual intake distribution of the population, the method developed by Iowa State University (ISU) was applied.

The ISU method was implemented using PC-SIDE software (version 1.0, Department of Statistics, Center for Agricultural and Rural Development, Ames, IA, USA). This program estimates usual nutrient and food intake distributions, moments, and percentiles. Whether the intake corresponds to the first 24 h DR from the initial interview or the telephone interview was considered in the adjustment of dietary data, stratifying by sex, age group, and cohorts (SRS or AMS).

To assess nutrient adequacy, individual usual intake (IUI) was compared to the current recommendations of adequate intake (AI) and reference intake range (RI) defined by EFSA [26] and according to UN-FAO [12], including accepted macronutrient distribution range (AMDR), which was set as a percentage of total energy intake (%E). The participants were divided into age groups established by EFSA and UN-FAO for a complete evaluation.

The results obtained were processed using different statistical methods. The Kolmogorov–Smirnoff normality test was used to determine the normality of distribution of the variables to decide between parametric or non-parametric analysis for comparison.

The comparison by sex and age group between cohorts (SRS and AMS) was performed by Mann–Whitney U-test. Kruskal–Wallis analysis was used to calculate differences among age groups within cohorts. A chi-squared test was used to evaluate adequacy differences between cohorts (SRS and AMS) and by age group. Linear correlation, collinearity test, and logistic regression analyses were applied to explore any influence of selected sociodemographic, anthropometrical, and physical activity variables on the nutrient intake of interest. Analyses of covariance (ANCOVA) were performed to evaluate what variables, e.g., anthropometry, socio-economic status, and geographic distribution, could influence the intakes of the main fatty acids. The level of significance was set at 5% (*p* < 0.05). All data analyses were performed using IBM SPSS 20.0 (IBM Corp., Armonk, NY, USA).

## 3. Results

### 3.1. Subjects’ Characteristics

A total of 1448 children ages 1 to <10 years were enrolled in the study; details of the subject characteristics; general, anthropometric, and socio-economic data have been published previously [36,49].

Total SRS represented 48.8% and AMS 51.1%. Three age groups stratified each cohort according to the stage of growth and development of the children: group 1 (toddlers), 1 to <3 years old (31.5%); group 2 (preschoolers), 3 to <6 years old (34.9%); and group 3 (school-aged), 6 to <10 years old (33.6%). The characteristics of both study cohorts are given in Table 1.

### 3.2. Dietary Lipid Profile and Distribution

Table 2 shows total lipid and main FA intake according to age group, and Supplementary Table S1 according to age group and sex. The SRS cohort reported a higher intake of total fat and FAs than the AMS cohort (*p* = 0.002): total fat: 59.77 vs. 54.94 g/day; SFAs: 21.23 vs. 18.46 g/day, MUFAs: 25.13 vs. 23.56 g/day; PUFAs: 7.16 vs. 6.60 g/day; and n-6 PUFA: 5.91 vs. 5.21 g/day. Nevertheless, the AMS cohort had higher n-3 intake (0.64 vs. 0.59 g/day, *p* = 0.002) and a lower n-6:n-3 ratio (8.38 vs. 10.14, *p* < 0.001).

When comparing AMS and SRS by age group, the children in AMS of the group 1 (1 < 3 years old) had a lower intake of total fat (43.20 vs. 47.34 g/day, *p* = 0.016) and SFAs (14.90 vs. 16.88 g/day, *p* < 0.001) than the same age group of the SRS. The children in AMS cohort group 2 also had a lower intake of SFAs (20.88 vs. 22.18 g/day, *p* = 0.038). Three age groups of children in the AMS cohort were found to have higher intake of n-3 (group 1: 0.58 vs. 0.49 g/day; group 2: 0.68 vs. 0.59 g/day; group 3: 0.71 vs. 0.66 g/day; *p* < 0.005). Finally, three age groups of children in the SRS cohort showed a higher n-6:n-3 ratio (group 1: 9.25 vs. 7.33; group 2: 10.13 vs. 9.21; group 3: 10.50 vs. 8.91; *p* < 0.005).

When analyzing data including and excluding misreporters, no significant differences were found (data not shown); for this reason, and following the EFSA recommendation, misreporters were not excluded from all the analyses made in the present study [41]. Supplementary Table S2 gives the total lipid and FA intake of plausible reporters.

Table 3 shows the distribution of reported intakes of total fat and main fatty acids as the percentage of contribution to the total energy intake (%EI) from two 24-h dietary recalls according to age group. The SRS cohort reported a higher percentage of contribution to the total energy intake from total fat (36.2% vs. 35.8%, *p* = 0.033) and SFAs (13.1% vs. 12.1%, *p* < 0.001) than the AMS cohort. On the contrary, the AMS cohort reported a higher percentage of contribution of the n-3 than the SRS cohort (0.4% vs. 0.4%, *p* < 0.001).

When comparing AMS and SRS by age group, the children in group 1 and group 2 of the SRS cohort had a higher percentage of contribution to the total energy intake of SFAs than their counterparts of the AMS cohort (group 1: 12.5% vs. 11.3%; group 2: 13.4% vs. 12.3%; *p* < 0.005). Children in groups 2 and group 3 of the AMS cohort had a higher percentage of contribution of PUFAs (group 2: 4.7 vs. 4.3; group 3: 4.8% vs. 4.3%; *p* < 0.005) than the same age groups of the SRS cohort. All the children in the AMS cohort had a higher percentage of contribution to the total energy intake of n-3 than the children in the SRS cohort (group 1: 0.4% vs. 0.3%; group 2: 0.4% vs. 0.3%; group 3: 0.4% vs. 0.4%; *p* < 0.005).

Table 4 shows the distribution of individual usual intakes of total fat and main fatty acids as the percentage of contribution to the total energy intake (%EI) from two 24-h dietary recalls according to age group. The SRS cohort reported a higher percentage of contribution to the total energy intake from total fat (36.9% vs. 36.5%, *p* = 0.008), SFAs (13.3% vs. 12.2%, *p* < 0.001), PUFAs (4.2% vs. 4.1%, *p* = 0.028), and n-6 (3.8% vs. 3.7%, *p* < 0.001). Nevertheless, the AMS cohort reported a higher percentage of contribution from n-3 than the SRS cohort (0.5% vs. 0.4%, *p* < 0.001).

When comparing AMS and SRS by age group, the children in the group 1 of the SRS cohort showed a higher percentage of contribution to total energy from total fat (36.7% vs. 35.2%, *p* < 0.001) and the children in the group 1 and group 3 from n-6 (group 1: 3.6% vs. 3.4%; group 3: 3.9% vs. 3.7%, *p* < 0.005) than the same age groups of the AMS cohort. All the children in the SRS have a higher percentage of contribution to total energy from SFAs than the pair group of the AMS cohort (group 1: 13.1% vs. 11.4%; group 2: 13.5% vs. 12.5%; group 3: 13.3% vs. 13.0%, *p* < 0.005). Besides, all children of the AMS cohort had a higher percentage of contribution to the total energy intake from n-3 than the peers age group of the SRS cohort (group 1:0.5% vs. 0.4%; group 2: 0.5% vs. 0.4%; group 3: 0.4% vs. 0.4%, *p* < 0.005). Finally, the group 2 and group 3 from the AMS cohort had a higher percentage of contribution to total energy from MUFAs than the pair group of the SRS cohort (group 2: 15.9% vs. 15.5%; group 3: 16.3% vs. 15.8%; *p* < 0.005).

The statistically significant differences between both cohorts (SRS and AMS) in the total fat, SFAs, PUFAs and n-6 intake are mainly due to the diet and in a minor proportion are influenced by the Nielsen area and the highest level of education achieved by one of the parents as main covariates (Table 5); n-3 differences between SRS and AMS were due only to diet.

### 3.3. Major Fatty Acid Profile and Distribution

Table 6 shows the intake of major FAs from the two cohorts (SRS and AMS) according to age group, and Supplementary Table S3 according to age group and sex. Intake of all FAs increased with age, and there were significant differences between cohorts. SRS had significantly higher intake of myristic acid (1.63 vs. 1.02 g/day), palmitic acid (10.45 vs. 7.85 g/day), oleic acid (21.73 vs. 17.74 g/day), LA (5.85 vs. 5.15 g/day), ALA (0.44 vs. 0.42 g/day), AA (0.06 vs. 0.05 g/day), and DPA (0.03 vs. 0.03 g/day) (*p* < 0.005). Higher intake of EPA and DHA (0.06 vs. 0.01 g/day and 0.09 vs. 0.02 g/day, respectively; *p* < 0.001) was reported for AMS.

There were differences by age group between the two cohorts. The three age groups of children in the the SRS cohort showed significantly higher intake of myristic, palmitic, stearic, palmitoleic, and oleic acid. Children in group 2 were found to have higher intake of AA and DPA (0.07 vs. 0.05 g/day and 0.04 vs. 0.03 g/day; *p* = 0.006 and *p* = 0.036, respectively). Children in group 3 showed higher intake of ALA and AA (0.49 vs. 0.45 g/day and 0.07 vs. 0.06 g/day; *p*= 0.033 and *p* = 0.029, respectively). Conversely, children in AMS cohort group 1 reported higher intake of ALA and DHA (0.38 vs. 0.35 g/day and 0.10 vs. 0.02 g/day; *p* = 0.039 and *p* < 0.001, respectively). Groups 2 and 3 had higher intake of EPA (group 2: 0.07 vs. 0.01 g/day; group 3: 0.11 vs. 0.01 g/day; *p* < 0.001) and DHA (0.09 vs. 0.02 g/day and 0.08 vs. 0.02 g/day; *p* < 0.001).

### 3.4. Adequacy of Lipid Profile According to European Food Safety Authority (EFSA) Recommendations

IUI for total fat, LA, and ALA was calculated to compare reported intake with EFSA recommendations. Table 7 shows the percentages of children meeting and not meeting the EFSA recommendations for the main FAs by cohort and age group and Supplementary Table S4 for the plausible energy reporters of the two cohorts.

Children below the EFSA recommendations could be at risk of EFAs and n-3 long-chain polyunsaturated fatty acids (LC-PUFA) deficiencies. In contrast, children above the recommendations do not exceed the tolerable upper intake limit (LA: 20%E; ALA: 3%E; EPA+DHA: 2%E) and are not at risk of any adverse effects due to toxicity.

For total fat, the SRS cohort had a higher percentage of children who met or were above EFSA recommendations (41.7% vs. 37.1% and 44.6% vs. 35%, respectively; *p* < 0.001). Regarding LA, this cohort had a higher percentage of children who met or were above the recommendations (26.4% vs. 24.4% and 16.1% vs. 10.9%, respectively; *p* = 0.004). The AMS cohort had a higher percentage of children who met or were above the recommendations for ALA (2.4% vs. 1.0% and 0.9% vs. 0%, respectively; *p* < 0.001) and EPA+DHA (2.6% vs. 2.4% and 38.2% vs. 24.2%; *p* = 0.004 and *p* < 0.001, respectively) than the SRS cohort. Lastly, there were differences by age group between both cohorts. For total fat, more children in the SRS cohort group 1 above the recommendations than the same AMS cohort age group (22.2% vs. 8.5%; *p* < 0.001).

For LA, more children met the recommendations in the SRS cohort group 1 and above the recommendations in group 3 than in the same AMS age groups (25.3% vs. 13.9% and 20.6% vs. 11.4%, respectively; *p* = 0.010). For ALA, more children in the SRS cohort group 1 were below the recommendations than in the AMS peer group (98.8% vs. 93.2%; *p* = 0.025). Finally, for EPA+DHA, more children above the recommendations in AMS groups 1, 2, and 3 than their SRS counterparts (group 1: 39.5% vs. 24.7%; group 2: 35.5% vs. 23.0%; group 3: 40.0% vs. 24.9%; *p* < 0.001).

### 3.5. Adequacy of Lipid Profile to the Food and Agriculture Organization of the United Nations (UN-FAO) Recommendations

IUIs for total fat, LA, and ALA were calculated to compare reported intake with the UN-FAO recommendations. Table 8 shows the percentages of children meeting and not meeting the UN-FAO recommendations for the main FAs by cohort and age group and Supplementary Table S5 according to the plausible reporters of the two cohorts.

Children below the FAO recommendations could be at risk for EFAs and n-3 LC-PUFA deficiencies. However, children above the recommendations do not exceed the tolerable upper intake limit (LA: 20%E; ALA: 3%E; EPA+DHA: 2%E); this means they are not at risk of adverse effects.

For total fat, more children in the SRS cohort met or were above the recommendations than in the AMS cohort (38.2% vs. 35.6% and 57.9% vs. 52.8%, respectively; *p* < 0.001). A significantly higher percentage of children in the AMS cohort met the recommendations for PUFAs than in the SRS cohort (21.5% vs. 11.2%; *p* < 0.001). Also, for EPA+DHA, more children in the AMS cohort met or were above the recommendations (12.0% vs. 5.0% and 42.0% vs. 27.3%, respectively; *p* < 0.001).

There were differences in total fat and EPA+DHA between AMS and SRS age groups. For total fat, more children were below the recommendations in AMS group 1 (29.3% vs. 16.0%; *p* < 0.001). Finally, for EPA+DHA, more children met or were above the recommendations in the three AMS age groups than their SRS peer groups (group 1: 13.3% vs. 4.9%; group 2: 11.5% vs. 4.5%; group 3: 10.8% vs. 5.3%; *p* < 0.005) (group 1: 39.5% vs. 28.4%; group 2: 46.2% vs. 29.5%; group 3: 40.0% vs. 24.9%; *p* < 0.005.

### 3.6. Individual Usual Intake of Total Fat and Main Fatty Acids and Family and Personal Factors

Table 9, Table 10, Table 11 and Table 12 show the odds ratios (ORs) and confidence intervals (CI) for intake equal to or higher than the median intake of PUFAs, n-3, and n-6, and the n-6:n-3 ratio relative to family and personal factors (covariates) in the SRS and AMS cohorts, respectively.

In the SRS cohort, girls had a significantly higher probability of having a MUFAs intake above the median (OR 1.42, 95% CI 1.01–1.99). Also in the SRS cohort, a parent with secondary education and the child’s z-BMI/age was related to the probability of having an intake above the median of SFAs (OR 1.62, 95% CI 1.03–2.53), n-6 (OR 1.58, 95% CI 1.02–2.44), and n-3 (OR 1.11, 95% CI 1.01–1.22). PAL reduced the odds of having a PUFAs intake above the median (OR 0.45, 95% CI 0.22–0.94).

In the AMS cohort, children of families with income >€2000 had a significantly lower probability of having an intake above the median of total fat (OR 0.48, 95% CI 0.27–0.87), MUFAs (OR 0.52, 95% CI 0.29–0.92), and n-3 (OR 0.56, 95% CI 0.35–0.89). On the contrary, one parent with secondary education increased the probability of having an n-6:n-3 ratio above the median (OR 1.56, 95% CI 1.02–2.39) and the child’s height for age z-score increased the probability of having a PUFAs intake above the median (OR 1.18, 95% CI 1.05–1.33).

For both SRS and AMS cohorts, groups 2 and 3 showed significantly less probability of having an intake of the considered fatty acid groups above the median. AMS group 2 and SRS groups 2 and 3 showed significantly less probability of having an intake of total fat, SFAs, MUFAs, PUFAs, n-3, and n-6 and n-6:n:3 ratio above the median.

### 3.7. Contribution of Food Groups to Total Lipid and Main Fatty Acid Intake

**Figure 1:**
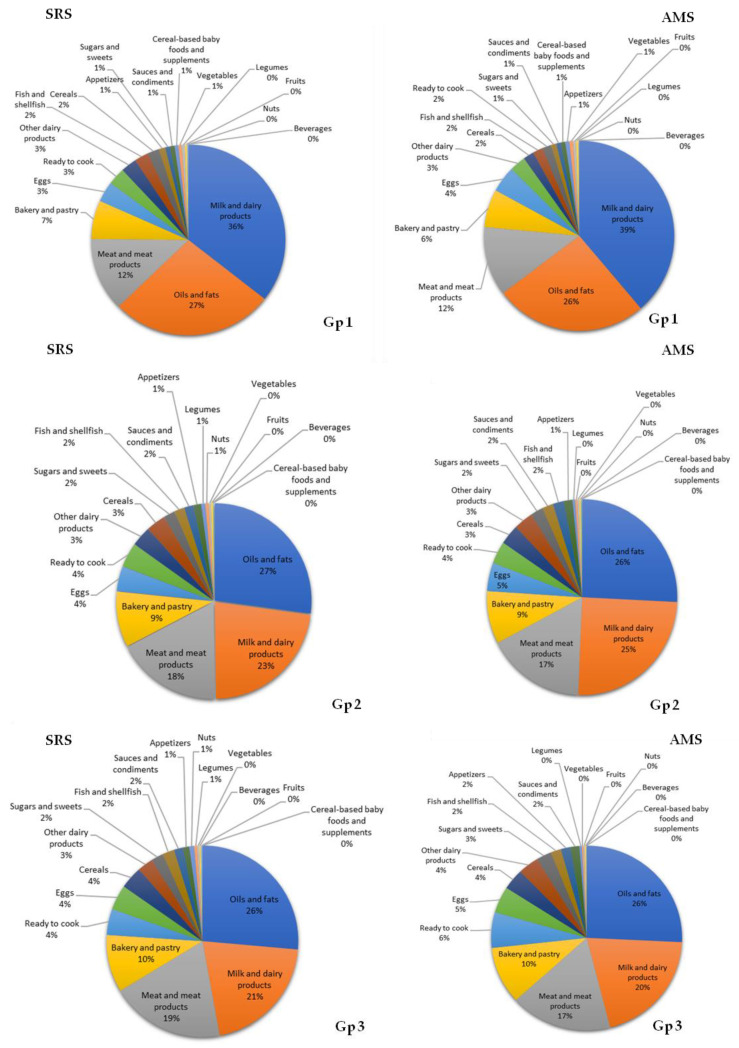
Contribution (%) of the 18 food groups to total fat intake in the EsNuPI study population (Spanish Reference Cohort (SRS) and Adapted Milk Consumers Cohort (AMS)) according to age group (**Gp 1**0 1 to <3 years; (**Gp 2**), 3 to <6 years; (**Gp 3**) 6 to <10 years).

The food groups that represent the most important sources of total fat intake in SRS and AMS group 1 were milk and dairy products (36% and 39%), oils and fats (27% and 26%), and meat and meat products (12% and 12%). This order changed in groups 2 and 3 considering SRS and AMS results, respectively: oils and fats (group 2: 27% and 26%; group 3: 26% and 26%), milk and dairy products (group 2: 23% and 25%; group 3: 21% and 20%), and meat and meat products (group 2: 18% and 17%; group 3: 19% and 17%) (Figure 1).

**Figure 2:**
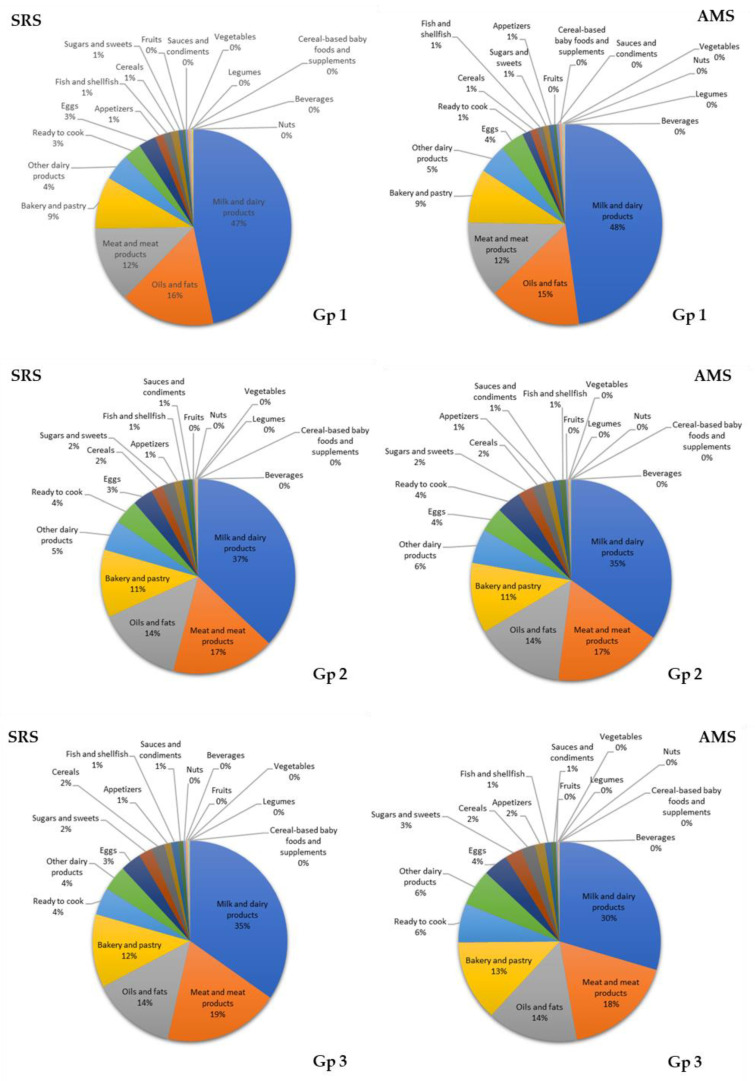
Contribution (%) of the 18 food groups to saturated fatty acids (SFAs) intake in the EsNuPI study population (Spanish Reference Cohort (SRS) and Adapted Milk Consumers Cohort (AMS)) according to age group (**Gp 1**), 1 to <3 years; (**Gp 2**), 3 to <6 years; (**Gp 3**) 6 to <10 years).

The main sources of SFAs in both SRS and AMS group 1 were milk and dairy products (47% and 48%), oils and fats (16% and 15%), meat and meat products (16% and 15%), and bakery and pastry (9% in both groups). In groups 2 and 3, the main sources of SFAs were milk and dairy products (group 2: 37% and 35%; group 3: 35% and 30% for both groups), meat and meat products (group 2: 17% for both; SRS group 3: 19%; and AMS group 3: 18%), and oils and fats (14% for groups 2 and 3 in both) (Figure 2).

The main sources of MUFAs for both SRS and AMS group 1 were oils and fats (46% and 44%), milk and dairy products (19% and 25%), and meat and meat products (14% and 13%). In groups 2 and 3 from both groups, the greatest contribution came from oils and fats (group 2: 41% and 38%; group 3: 39% and 37%), meat and meat products (group 2: 18% and 19%; group 3: 20% and 17%), and milk and dairy products (group 2: 15% and 19%; group 3: 14% and 17%) (Supplementary Figure S1).

As for PUFAs intake, oils and fats (33% and 28%), milk and dairy products (19% and 26%), and meat and meats products (14% and 14%) were the highest contributors in group 1 of both groups. In groups 2 and 3 of both cohorts, the main contributors were oils and fats (group 2: 33% and 31%; group 3: 31% and 31%), followed by meat and meat products (group 2: 19% and 17%; group 3: 20% and 19%), and bakery and pastry (group 2: 8% and 8%; group 3: 8% and 8%) (Supplementary Figure S2).

Specifically, for n-3 fatty acid intake, in both SRS and AMS group 1, the main contributors were milk and dairy products (23% and 21%), meat and meat products (15% and 18%), fish and shellfish (15% and 15%), and oils and fats (14% and 13%). However, in both SRS and AMS groups 2 and 3 n-3 fatty acid intake consisted of milk and dairy products (group 2: 23% and 22%; group 3: 23% and 23%), fish and shellfish (group 2: 17% and 18%; group 3: 16% and 18%), and meat and meat products (group 2: 15% and 17%; group 3: 14% and 16%) (Supplementary Figure S3).

The food groups that represent the most important sources of n-6 intake in SRS and AMS group 1 were oils and fats (37% and 31%), milk and dairy products (20% and 25%), and meat and meat products (16% and 15%). In groups 2 and 3 of both groups, the primary contributors were oils and fats (group 2: 34% and 34%; group 3: 32% and 33%), meat and meat products (group 2: 20% and 18%; group 3: 20% and 19%), and bakery and pastry (8% for both groups from both groups) (Supplementary Figure S4).

**Figure 3:**
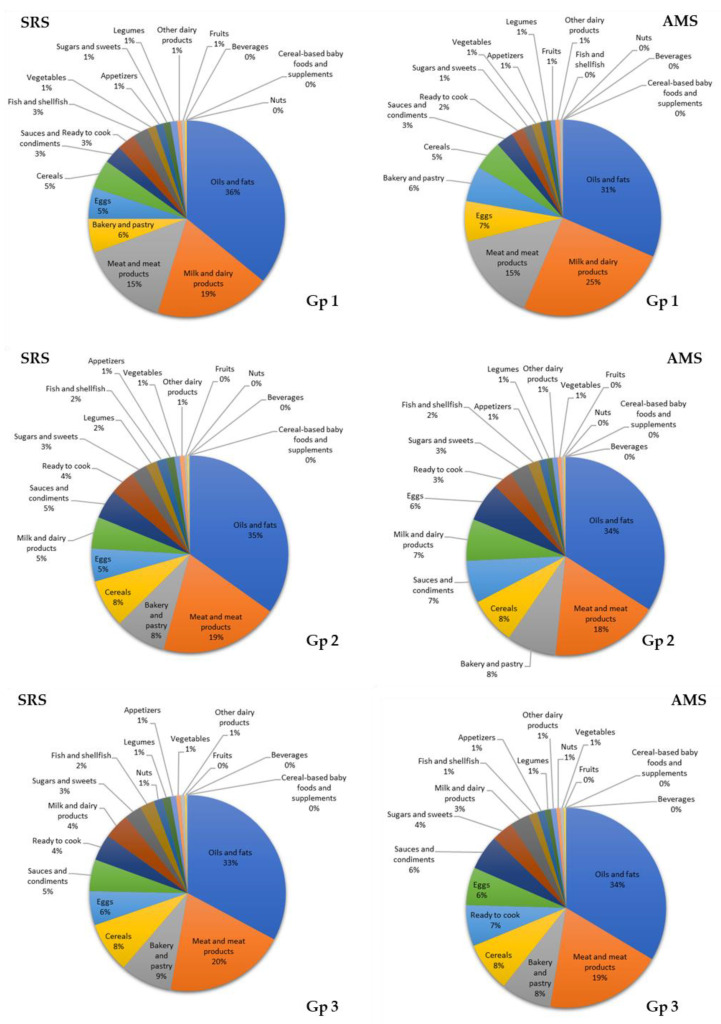
Contribution (%) of the 18 food groups to linoleic acid (LA) intake in the EsNuPI study population (Spanish Reference Cohort (SRS) and Adapted Milk Consumers Cohort (AMS)) according to age group ((**Gp 1**), 1 to <3 years; (**Gp 2**), 3 to <6 years; (**Gp 3**), 6 to <10 years).

Oils and fats (36% and 31%), milk and dairy products (19% and 25%), and meat and meat products (15% and 15%) were the greatest contributors of LA in group 1 from both cohorts. In groups 2 and 3 from both cohorts, the major contributors of LA were oils and fats (group 2: 35% and 34%; group 3: 33% and 34%), meat and meat products (group 2: 19% and 18%; group 3: 20% and 19%), and bakery and pastry (group 2: 8% and 8%; group 3: 9% and 8%) (Figure 3).

**Figure 4:**
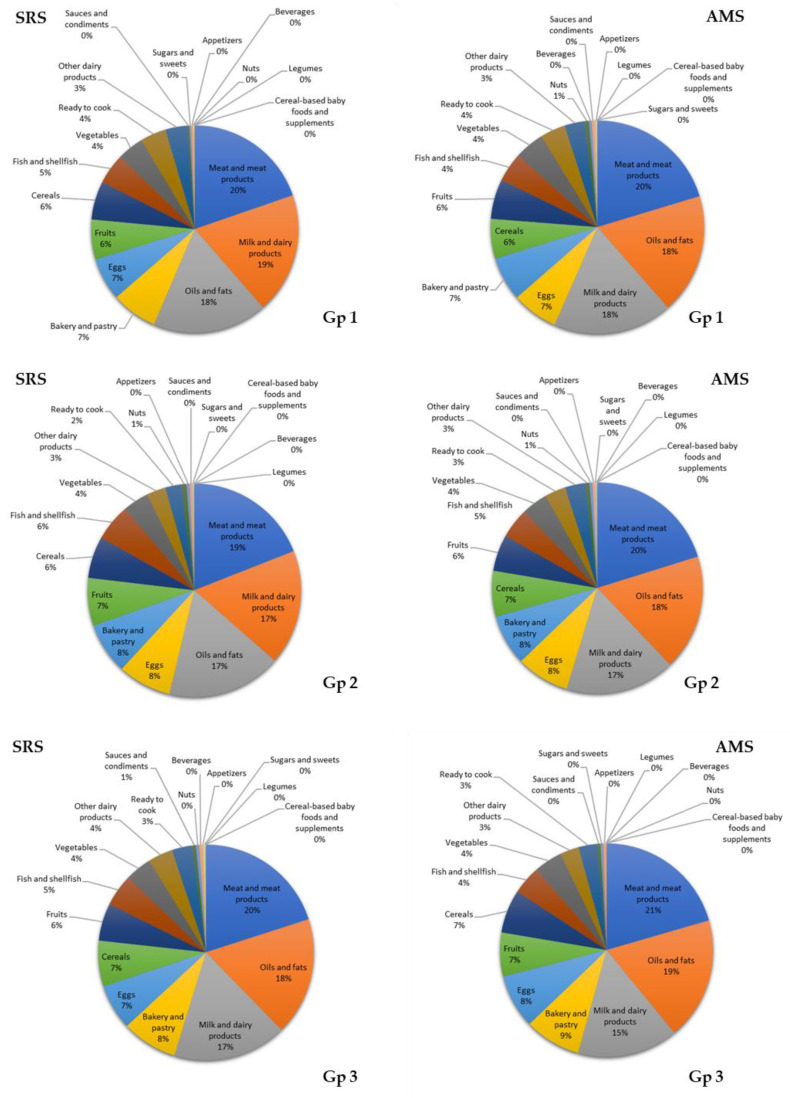
Contribution (%) of the 18 food groups to α-linolenic acid (ALA) intake in the EsNuPI study population (Spanish Reference Cohort (SRS) and Adapted Milk Consumers Cohort (AMS)) according to age group ((**Gp 1**), 1 to <3 years; (**Gp 2**), 3 to <6 years; (**Gp 3**) 6 to <10 years).

In the groups, 1 and 2 from both cohorts, meat and meat products (group 1: 20% and 10%; group 2: 19% and 20%), milk and dairy products (group 1: 19% and 18%; group 2: 17% and 17%), and oils and fats (group 1: 18% and 18%; group 2: 17% and 18%) were the main contributors of ALA. Moreover, in group 3 from both cohorts, the greatest contributors were meat and meat products (20% and 21%), oils and fats (18% and 19%), and milk and dairy products (17% and 15%) (Figure 4).

**Figure 5:**
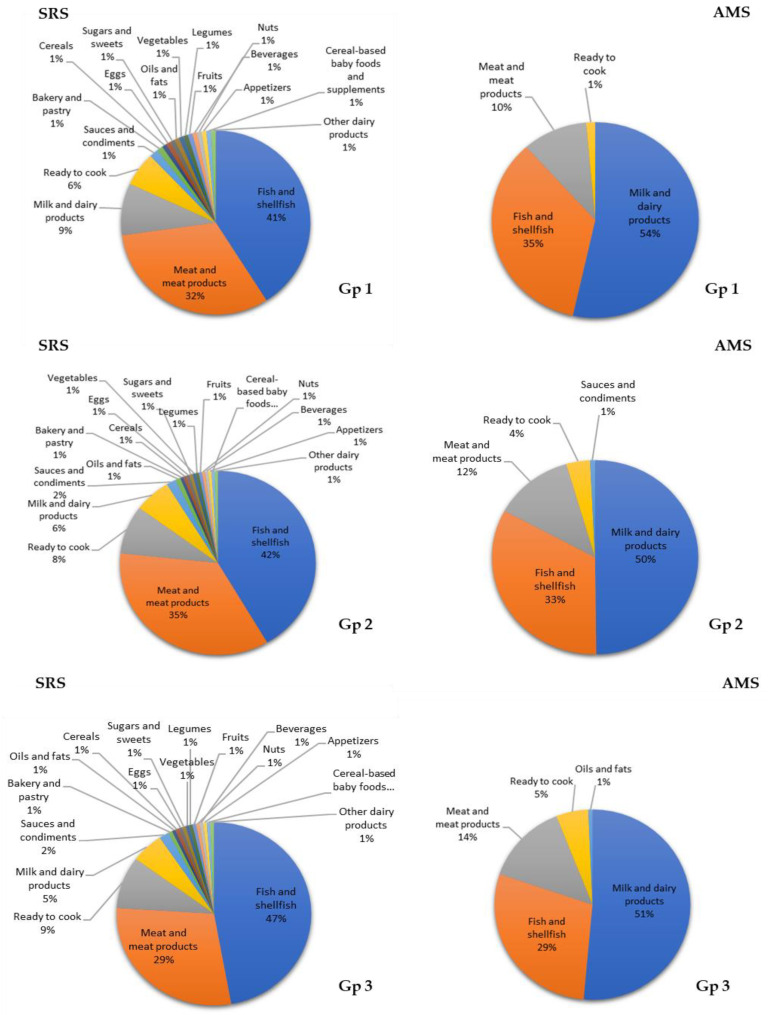
Contribution (%) of the 18 food group to docosahexaenoic acid (DHA) intake in the EsNuPI study population (Spanish Reference Cohort (SRS) and Adapted Milk Consumers Cohort (AMS)) according to age group ((**Gp 1**), 1 to <3 years; (**Gp 2**), 3 to <6 years; (**Gp 3**), 6 to <10 years).

For DHA intake, in the SRS the main food sources in the three age groups were the fish and shellfish (group 1: 41%; group 2: 42%; group 3: 47%) meat and meat products (group 1: 32%; group 2: 35%; group 3: 29%), and milk and dairy products (group 1: 9%; group 2: 6%; group 3: 5%). On the contrary, for the AMS the main contributors of DHA in the three age groups were milk and dairy products (group 1: 54%; group 2: 50%; group 3: 51%), fish and shellfish (group 1: 35%; group 2: 33%; group 3: 29%), and meat and meat products (group 1: 10%; group 2: 12%; group 3: 14%) (Figure 5).

Finally, for the EPA intake, in the group 1, 2 and 3 from both cohorts, milk and dairy products (group 1: 46% and 50%; group 2: 45% and 45%; group 3: 49% and 48%), fish and shellfish (group 1: 30% and 31%; group 2: 36% and 33%; group 3: 31% and 35%) and meat and meat products (group 1: 9% and 10%; group 2: 7% and 13%; group 3: 8% and 10%) were the main contributors of EPA (Supplementary Figure S5).

## 4. Discussion

The EsNuPI study is the most recent survey in Spain that provides current data about dietary intake and food sources of total fat and major FAs in one representative cohort of Spanish children and one cohort of adapted milk consumers, both cohorts aged 1 to <10 years and living in urban areas. It also evaluates the influence of different factors (sociodemographic, anthropometric, physical activity, educational level, etc.) on energy and nutrient intake.

The present study shows that in the EsNuPI population, there is a high intake of total fat and SFAs regardless of the considered age group and low intake of EFAs (LA and ALA) and LC-PUFA of the n-3 series, mainly DHA. Furthermore, the consumption of adapted milk contributes to better adherence to EFSA and UN-FAO recommendations for intake of total fat, SFAs, EFAs, PUFAs, and EPA+DHA. However, a high percentage of children in the survey did not reach the intake recommendations for EFAs and PUFAs. In the study population, for both considered cohorts (SRS and AMS), milk and dairy products, oils and fats, and meat and meat products were the main sources of total fat, SFAs, MUFAs, and PUFAs, and oils and fats, milk and dairy products, meat and meat products, and fish were the main sources of major FAs. The most important personal factor that influenced the consumption of fat and FAs in both cohorts of our study was age; increasing age seemed to be a protective factor for consumption of fat and major FAs above the median.

Our results showed differences in the intakes of total fat, SFAs, PUFAs, and n-6 between the SRS and AMS cohorts, these differences are mainly due to the diet and, as influencing factors, by living area (Nielsen area) and by the highest level of education achieved by one of the parents. Differences in n-3 intakes between the two cohorts were due only to the diet and did not show any influence by covariates such as Nielsen area, the highest level of education achieved by one of the parents, and the socioeconomic status. Differences in lipid profile and main fatty acids in children’s intake by socio-economic factors have been reported by other authors [50,51].

Knowledge of the quality of the diet and other factors that influence children’s nutritional status is important, and further research is needed to evaluate them in the EsNuPI study population.

### 4.1. Lipid Profile and Adequacy

We evaluated the adequacy of the intake of FAs in the EsNuPI study population using the latest EFSA and FAO/WHO recommendations to be able to compare our results with European and International recommendations. However, it is important to note that the percentage of children below the recommendations may be at risk for FA deficiencies that could result in growth and development problems. On the contrary, the percentage of children above the recommendations do not exceed the tolerable upper intake limit (LA: 20%E; ALA: 3%E; EPA+DHA: 2%E), hence, not physiologically changes are expected.

When comparing with the recommendations for total fat intake, our results show that in both cohorts (SRS and AMS) the median intake of total fat was slightly higher. However, when analyzing at the individual level, a high percentage of children had a fat intake above the recommendations of both entities, with SRS showing a higher percentage (44.6%); 27.9% of children in the SRS cohort and 13.7% in the AMS cohort had fat intake below the recommended levels. Therefore, there is a possibility that these children had an inadequate intake of fat-soluble vitamins. Indeed, our cohort has reported that a large percentage of the EsNuPI population does not meet the recommended intake of vitamin D [49].

Also, we observed that total fat and FA intake increased with age in SRS and AMS cohorts. Similarly, Keim [52] reported that mean daily intake of n-6 PUFAs and EPA varied by age in US children aged 12–60 months; children 12–24 months of age had lower average intake than children aged 49–60 months and the lowest n-6:n-3 ratio, adjusting for energy intake.

In accordance with previous studies [4,7,53], a high intake of SFAs was observed in the Spanish pediatric population, with both cohorts showing a high median intake of SFAs (12.87% of TCV in SRS and 12.08% in AMS). The majority of children above the recommended amount of <8%E of SFAs established by the UN-FAO; nevertheless, the AMS cohort had a lower percentage of children above and a higher percentage meeting the recommendations than the SRS cohort.

With regard to n-3 and n-6 FAs, a high n-6:n-3 ratio is associated with weight gain and the promotion of pathogenesis of many diseases, including CVD, inflammatory, and autoimmune diseases [54]. In our study, children in the AMS cohort had a higher intake of n-3 (0.64 vs. 0.59 g/day) and a lower n-6:n-3 ratio (8.38 vs. 10.14). Guesnet et al. found that in a French population of 1500 children (3–10 years) and adolescents (11–17 years), inadequate consumption of n-6 and n-3 was observed, generating an unbalanced n-6:n-3 ratio [55].

Regarding the DPA, it is the least studied LC-PUFA, compared to its counterparts EPA and DHA. Hence, there was insufficient data to produce specific recommendations. However, DPA is of current interest both for its ability to increase EPA and DHA tissue status, and for its specific or shared biological effects. Moreover, DPA is the most abundant LC-PUFA in the brain after DHA, and it could be specifically beneficial for the elderly neuroprotection and early-life development [56].

EFAs and PUFAs are necessary for healthy neuronal growth and maturation of the developing human brain, retina, and other organs. EFA deficiency results in clinical visual and neurological abnormalities and poor growth [57]. Nevertheless, the role of EFAs and PUFAs in cognition, immune function, and cardiovascular health is controversial. Dietary interventions suggest that EFAs may affect CVD risk factors in children in a similar way to adults [58]. Over the last years, there have been inconclusive systematic reviews and meta-analyses of the association between PUFAs and brain development, intelligence quotient scores, and disease modulation, such as the risk for allergy-related disorders [59,60].

Our findings of EPA+DHA intake are in line with studies from other countries that indicate inadequate intake of these PUFAs [61,62,63]. Our results show a low median intake; hence a small percentage of children met the EFSA and UN-FAO recommendations. According to EFSA, SRS mean intake of EPA+DHA was ~45% below AI, and that of AMS was ~28% below AI. AMS showed a higher percentage of children meeting and above the recommendations for PUFAs and EPA+DHA than SRS. Consumption of adapted milk guarantees n-3 PUFAs intake. This is in accordance with Ghisolfi’s study [64], which evaluated the nutritional adequacy of diets in early childhood as a function of intake of cow’s milk or growing-up milk (GUM) by children from 1 to 2 years of age. Cow’s milk consumption (≥250 mL/day) entails the risk of insufficient ALA, iron (Fe), and vitamins C and D, while the use of GUM (≥250 mL/d) significantly reduces the risk. We must keep in mind that these differences may be influenced by the composition of the formula consumed. Therefore, this shows that despite the observed differences between the lipid profiles of different infant formulas, using them is a viable strategy for child development [65]. However, in some cases, the composition of adapted milk could be inappropriate, when it is high in protein, fats, and carbohydrates, and even added sugars; this is due to the lack of standards and health effect studies.

In this sense, the European Society for Pediatric Gastroenterology Hepatology and Nutrition (ESPGHAN) Committee on Nutrition suggests that, based on the available evidence, the routine use of formula (adapted milks) for children in the first three years of life is unnecessary. However, it could be used as part of a strategy to increase the intake of Fe, vitamin D, and n-3 PUFA and decrease the intake of protein compared with unfortified cow’s milk [66]. During the nutritionally vulnerable period of 1 to 3 years of age, nutrient intake is often inadequate due to an unbalanced diet. The consumption of adapted milks may help infants and children at risk of nutrient deficiencies to meet their nutritional requirements. Moreover, protein, sodium, and vitamin A intakes often remained above the EFSA dietary reference values (DRVs), and DHA, ARA, and vitamin D remained below [67].

In the Vieux study [68], dietary changes and nutritional adequacy in 1147 UK children aged 12 to 18 months who consumed adapted milks and or supplements or regular milk were evaluated. Nutritional adequacy with repertoire foods alone was ensured for only one child in the group, who consumed neither adapted milk nor supplements, against 74.4% of children who did consume them. Consequently, adapted milks can be used to improve nutritional adequacy. However, other key strategies for optimizing dietary intake include the promotion of a varied healthy diet and the use of fortified foods and supplements.

### 4.2. Contributions of Food Groups to Fat and Major Fatty Acid Consumption

In the present study, the top three food sources of total fat, SFAs, MUFAs, PUFAs, n-3 fatty acid, and n-6 fatty acid among children were milk and dairy products, oils and fats, and meat and meat products. However, the relative importance of those food sources could differ according to age and population. Vieux [68] showed that smaller food changes in the food repertoire were required to ensure nutritional adequacy when adapted milks and supplements were initially consumed than when they were not. Therefore, in the whole group, increasing adapted milk and supplement consumption was the shortest way to cover the EFSA nutrient requirements for UK children. Eussen et al. [69] showed that for infants and young children 12 to 18 years old in the UK Diet and Nutrition Survey, replacing habitual cow’s milk intake by an equal volume of 300 mL of adapted milk led to nutritional intake more in line with recommendations. Besides, Lovell [53], in a multi-center, double-blinded, randomized controlled trial of children receiving reduced-protein growing-up milk (GUMLi) or unfortified CM, assessed the probability of adequate nutrient intake (PANDiet) at months 7, 8, 10, and 11 post-randomization, showing that total PANDiet scores were significantly higher in the GUMLi group, indicating better diet quality.

Comparing the main food group contributors to nutrient intake is difficult because food groupings often differ between countries. However, in European studies, milk and dairy products, meat and meat products, and oils and fats provide a large contribution to total fat, SFAs, MUFAs, and PUFAs among older children [8,70,71]. By contrast, in the US NHANES 2011–2014 study, for children 2–18 years of age (*n* = 5876), milk, sweet bakery products, and pizza were the top three food sources of SFAs [72].

As described by others, the main sources of n-3 and DHA are fish, milk and dairy products, and meat and meat products [62,73]. We observe that in our study population, the contribution of fish to n-3 intake increases very little with age. These trends show the difficulty of following the right Mediterranean dietary pattern by the pediatric population.

Food consumption and habits in Spain have experienced a significant change in the last few years (social and economic changes), leading to a transition in dietary patterns and lifestyles that are moving away from the traditional Mediterranean diet showing a tendency towards an increasingly “westernized” diet that comprise a higher intake of animal food sources instead of plant-based products such as legumes, nuts, fruits, and vegetables [4,7,27,61]. These changes could lead to an inadequate intake of energy and nutrients, which in turn might have potential adverse effects on children’s health and predispose them to the risk of development of non-communicable diseases [47,52].

In general, in our study, milk and dairy products played a primary role as a source of total fat and SFAs, followed by meat and meat products, and oils and fats. SFAs differ based on their carbon chain length and are categorized as short-, medium-, long-, and very long-chain fatty acids [74]. Dairy fats are the major food sources of short-chain and red meat, dairy fats, and plant oils of medium and long-chain SFAs. The food sources of SFAs contain different percentages of diverse fatty acids besides other nutrients that can influence their physiological and biologic effects [74,75]. Also, SFAs are classified according to the presence or absence of methyl branches on the carbon chain. Branched-chain SFAs are found mainly in dairy, beef, and other foods derived from ruminants. Moreover, in experimental animal studies, branched-chain fatty acids change the composition of the microbiota and could take a role in normal colonization [76].

Health effects of dietary SFAs have been studied in the last years [77,78], and they are generally thought to have detrimental effects on health. However, recent controversies exist on the effects of SFAs on obesity, type 2 diabetes, and CVD [79]. SFAs are found in a wide diversity of foods that vary in origin, chemical composition, and metabolic structure, resulting in different physiological and biologic effects [30,80]. Indeed, observational studies show different associations for SFAs of varying physical, chemical, and metabolic structures, hence supporting different effects of diverse SFAs on blood lipids, glucose-insulin homeostasis, insulin resistance, and diabetes. Furthermore, various cohort studies demonstrated that the replacement of fat with carbohydrates was not associated with a lower risk of CVD and could be associated with an increase in mortality [81,82]. Besides, in the PURE (Prospective Urban Rural Epidemiological) study (*n* = 135,000 participants without CVD) from 18 countries on 5 continents, the high consumption of all types of fat (saturated, monounsaturated, and polyunsaturated) was associated with a lower risk of death and with a neutral association of the development of CVD. By contrast, a diet with a high intake of carbohydrates was associated with a higher risk of death, but not with the risk of CVD [83].

The health benefit of fats is not just because of their content in SFA; it is also a result of the various components in the food, commonly called the “food matrix” [75,76]. Dairy is the major source of SFA in many countries, and several dietary guidelines recommend low-fat or fat-free versions of dairy foods to limit SFA intake. However, food-based meta-analyses find that cheese and yogurt intakes are inversely associated with CVD risk, and full-fat dairy could be protective against type 2 diabetes [32,33]. Furthermore, the intake of processed meat has been associated with an increased risk of CVD. On the contrary, the intake of unprocessed red meat is not, which indicates that the SFA content of meat is not likely to be responsible for this association [81].

Furthermore, the influence of family and personal factors on total fat and main FA intake was observed in this study. In SRS, secondary education of one of the parents and the child’s z-BMI/age were associated with the probability of being above SFAs and n-3 intake, while in AMS, secondary education of one parent increased the likelihood of being above the n-6:n-3 ratio and children’s height for age z-score of being above PUFAs intake. In Southern Ghanaian children 2–6 years of age, the EFAs did not differ between stunted and non-stunted children and were not associated with z-height/age or z-weight/age [84].

Complementary foods frequently have low concentrations of DHA and AA, and this is most significant in low-income countries, so in the age range of 6–36 months, the risk of EFA and DHA deficiency is very high and relates to low national income.

Future research directions should encompass in-depth assessments of specific cognitive outcomes, immune function, and disease incidence, as well as sources of experimental variability such as the status of fatty acid desaturase polymorphisms [85] to establish recommendations for dietary patterns that provide a healthy fat intake profile. Moreover, it is important to know the roles of different foods in this pattern, especially the types of milk, and future long-term controlled longitudinal studies with biomarker measurement should be conducted.

The environmental impact of infant formulas is very similar to cow’s milk production due to the lower percentage of milk and milk fat used in the process, besides the inclusion of vegetable oils and some raw materials that are reused [86].

### 4.3. Strengths and Limitations

The greatest strength of this research is that it is the first study to evaluate a representative cohort of Spanish children aged 1 to <10 years and one cohort of children who consume adapted milk, an age group for which there is scarce information. Nevertheless, reported bias might have influenced the results of the questionnaire in general. Therefore, following the EFSA recommendations, under- and over-reporters were identified in this study and were analyzed separately. Besides that, the two 24 h DRs do not reflect an individual’s usual intake. Consequently, the observed nutrient intake was transformed into the usual intake using the Nusser method. Furthermore, a potential limitation should be considered that we analyzed children living in urban areas but not in rural areas. Nevertheless, children living in urban areas represent 52.6% of the total Spanish child population aged one to <10 years.

## 5. Conclusions

This study is the first to estimate total fat and fatty acid intake in a representative cohort of Spanish children aged 1 to <10 years living in urban areas compared with a cohort of adapted milk consumers in this age range. Our findings show that our study population were above the SFAs intake recommendations but did not meet those for EFAs (LA and ALA) and long-chain PUFAs of the n-3 series, mainly DHA. According to our research, the consumption of adapted milk could be one of the main factors associated with better nutritional adherence to the recommendations of total fat, SFAs, EFAs, PUFAs, and resulted in being the main factor associated with better adherence to the recommendations of n-3 intakes.

Milk and dairy products, oils and fats, and meat and meat products played principal roles as sources of total lipids and FAs in the study population for both the Spanish reference cohort (SRS) and adapted milk consumers cohort (AMS).

Knowledge of dietary intake and food sources of total fat and FAs in children could help in the design and promotion of effective and practical age-appropriate guidelines to increase the consumption of EFA- and n-3-PUFA-rich foods in their diet. A longitudinal study at the national level with a larger sample stratified by region and socioeconomic factors, and ideally including specific blood biomarkers of nutrient intakes, is needed to confirm these results, to promote the appropriate consumption of foods, and to stimulate or modify milk and dairy fortification.

## Figures and Tables

**Table 1:** Distribution of studied cohorts in the Nutritional Study in the Spanish Pediatric Population (EsNuPI) (*n* = 1448).

EsNuPI Study Population		Whole Population	Spanish Reference Cohort (SRS)	Adapted Milk Consumers Cohort (AMS)
		*n* = 1448	*n* = 707	*n =* 741
Sex	Boys	728	357	371
	Girls	720	350	370
Age (years)	Group 1 1 to <3	456	162	294
	Group 2 3 to <6	506	244	262
	Group 3 6 to <10	486	301	185

Distribution of studied cohorts in EsNuPI study within-subjects with complete data of two 24 h dietary recalls and complete information on variables of interest.

**Table 2:** Total lipid and main fatty acids intake from two 24 h dietary recall for two cohorts of the Nutritional Study in the Spanish Pediatric Population (EsNuPI) according to age group (*n* = 1448).

**Spanish Reference Cohort (SRS)**																	
**(g)**	**Total** ***n* = 707**				**1 to <3 years** ***n* = 162**				**3 to <6 years** ***n* = 244**				**6 to <10 years** ***n* = 301**				***p***
	**Mean**	**SD**	**Median**	**IQR**	**Mean**	**SD**	**Median**	**IQR**	**Mean**	**SD**	**Median**	**IQR**	**Mean**	**SD**	**Median**	**IQR**	
**Total Fat**	61.31	20.39	59.77	24.86	49.60	16.84	47.34 ^a^	22.89	60.83	17.89	60.34 ^b^	20.73	68.00	21.16	64.77 ^c^	27.29	<0.001
**SFAs**	22.24	8.47	21.23	10.75	17.58	7.81	16.88 ^a^	8.53	22.43	7.20	22.18 ^b^	9.44	24.59	8.77	23.45 ^b^	10.60	<0.001
**MUFAs**	25.38	9.53	25.13	11.38	19.19	8.75	19.25 ^a^	11.56	25.56	8.21	25.29 ^b^	10.33	28.57	9.33	27.62 ^c^	11.83	<0.001
**PUFAs**	7.79	3.96	7.16	4.56	5.66	3.79	5.15 ^a^	4.16	7.72	3.41	7.16 ^b^	3.83	8.99	3.99	8.17 ^c^	4.21	<0.001
**n-3**	0.65	0.33	0.59	0.35	0.53	0.28	0.49 ^a^	0.30	0.63	0.27	0.59 ^b^	0.33	0.73	0.37	0.66 ^c^	0.37	<0.001
**n-6**	6.56	3.58	5.91	3.99	5.09	3.28	4.51 ^a^	3.51	6.44	3.25	5.91 ^b^	3.77	7.44	3.73	6.75 ^c^	4.01	<0.001
**n-6:n-3**	11.47	7.38	10.14	6.09	11.94	11.24	9.25 ^a^	6.36	11.14	5.31	10.13 ^a,b^	5.95	11.48	6.16	10.50 ^b^	5.81	<0.001
**Adapted Milk Consumers Cohort (AMS)**																	
**(g)**	**Total** ***n* = 741**				**1 to <3 years** ***n* = 294**				**3 to <6 years** ***n* = 262**				**6 to <10 years** ***n* = 185**				***p***
	**Mean**	**SD**	**Median**	**IQR**	**Mean**	**SD**	**Median**	**IQR**	**Mean**	**SD**	**Median**	**IQR**	**Mean**	**SD**	**Median**	**IQR**	
**Total Fat**	56.64	19.42	54.94 *	27.42	45.98	15.47	43.20 ^a,^*	20.71	61.22	18.64	61.12 ^b^	26.85	67.09	17.90	64.90 ^c^	24.44	<0.001
**SFAs**	19.28	7.47	18.46 *	10.48	15.18	5.73	14.90 ^a,^*	7.91	21.08	7.05	20.88 ^b,^*	10.57	23.25	7.34	22.00 ^c^	9.75	<0.001
**MUFAs**	23.88	9.30	23.56 *	12.14	18.67	8.14	18.38 ^a^	11.12	26.18	8.46	25.40 ^b^	12.10	28.88	8.08	28.00 ^c^	11.26	<0.001
**PUFAs**	7.23	3.61	6.60 *	4.36	5.17	2.70	4.90 ^a^	3.57	8.20	3.53	7.50 ^b^	4.67	9.10	3.37	8.51 ^c^	4.38	<0.001
**n-3**	0.70	0.33	0.64 *	0.34	0.61	0.29	0.58 ^a,^*	0.29	0.72	0.32	0.68 ^b,^*	0.36	0.79	0.37	0.71 ^b,^*	0.37	<0.001
**n-6**	5.89	3.12	5.21 *	3.82	4.54	2.39	4.20 ^a^	2.75	6.55	3.31	5.62 ^b^	4.08	7.12	3.08	6.53 ^c^	3.99	<0.001
**n-6:n-3**	9.13	4.87	8.38 *	5.29	8.11	5.36	7.33 ^a,^*	4.54	9.82	4.49	9.21 ^b,^*	5.33	9.76	4.27	8.91 ^b,^*	4.75	<0.001

SFAs, saturated fatty acids; MUFAs, monounsaturated fatty acids; PUFAs, polyunsaturated fatty acids; n-3, omega-3 fatty acids; n-6, omega-6 fatty acids. Average gram intake values from two 24 h dietary recalls were used. Results are expressed as mean, standard deviation (SD), median, and interquartile range (IQR). Mann–Whitney U-test was used to evaluate differences by total and age group between SRS and AMS cohorts (significant differences are marked with an asterisk in the median values of AMS). Kruskal–Wallis test was used to calculate differences among age groups within cohorts (significant differences are marked with superscript letters in median values of each age group). *p*-values for this test are included in the last column of the table. *p*-value < 0.05 was considered statistically significant.

**Table 3:** Distribution of reported intakes of total fat and main fatty acids as a percentage of the total energy intake (%EI) from two 24-h dietary recalls from the Nutritional Study in the Spanish Pediatric Population (EsNuPI) according to age group (*n* = 1448).

Spanish Reference Cohort (SRS)						Adapted Milk Consumers Cohort (AMS)				
	Total *n* = 707	1–<3 years *n* = 162	3–<6 years *n* = 244	6–<10 years *n* = 301	*p*	Total *n* = 741	1–<3 years *n* = 294	3–<6 years *n* = 262	6–<10 years *n* = 185	*p*
	%	%	%	%		%	%	%	%	
Total Fat	36.2	36.1	36.1	36.6	0.56	35.8	34.8 ^a,^*	36.3 ^b^	37.0 ^b^	<0.001
SFAs	13.1	12.5	13.4	13.0	0.101	12.1 *	11.3 ^a,^*	12.3 ^b,^*	12.7 ^b^	<0.001
MUFAs	14.9	13.9 ^a^	15.1 ^b^	15.2 ^b^	<0.001	15.2	13.9 ^a^	15.4 ^b^	15.9 ^b^	<0.001
PUFAs	4.2	3.5 ^a^	4.3 ^b^	4.5 ^b^	<0.001	4.3	3.6 ^a^	4.7 ^b,^*	4.8 ^b,^*	<0.001
n-3	0.4	0.3	0.3	0.4	0.71	0.4 *	0.4 ^a,^*	0.4 ^b,^*	0.4 ^a,b,^*	0.030
n-6	3.5	3.4 ^a^	3.5 ^a,b^	3.6 ^b^	0.016	3.5	3.3 ^a^	3.6 ^b^	3.7 ^b^	<0.001

SFAs, saturated fatty acids; MUFAs, monosaturated fatty acids; PUFAs, polyunsaturated fatty acids; n-3, omega-3 fatty acids; n-6, omega-6 fatty acids. Results are expressed as the median percentage of contribution to the total energy intake. Mann–Whitney U-test was used to evaluate differences by total and age group between SRS and AMS (significant differences are marked with an asterisk [*] in median values of the AMS cohort). Kruskal–Wallis test was used to calculate differences among age groups within cohorts (significant differences are marked with superscript letters in the median values of each age group). *p*-values for this test are included in the last column. *p*-value < 0.05 was considered statistically significant.

**Table 4:** Distribution of Individual Usual Intakes of total fat and main fatty acids as a percentage of the total energy intake (%EI) from two 24-h dietary recalls from the Nutritional Study in Spanish Pediatric Population (EsNuPI) according to age group (*n* = 1448).

	Spanish Reference Cohort (SRS)				*p*	Adapted Milk Consumers Cohort (AMS)				*p*
	Total *n* = 707	1–<3 Years *n* = 162	3–<6 Years *n* = 244	6–<10 Years *n* = 301		Total *N* = 741	1–<3 Years *N* = 294	3–<6 Years *n* = 262	6–<10 Years *n* = 185	
	%	%	%	%		%	%	%	%	
Total Fat	36.9	36.7	36.8	37.4	0.44	36.5 *	35.2 ^a,^*	36.8 ^b^	37.6 ^b^	<0.001
SFAs	13.3	13.1	13.5	13.3	0.27	12.2 *	11.4 ^a,^*	12.5 ^b,^*	13.0 ^b,^*	<0.001
MUFAs	15.4	14.5 ^a^	15.5 ^b^	15.8 ^b^	<0.001	15.6	14.3 ^a^	15.9 ^b,^*	16.3 ^b,^*	<0.001
PUFAs	4.2	3.9 ^a^	4.2 ^b^	4.2 ^b^	0.001	4.1 *	3.9 ^a^	4.3 ^b^	4.2 ^c^	<0.001
n-3	0.4	0.4 ^a^	0.4 ^a^	0.4 ^b^	0.001	0.5 *	0.5 ^a,^*	0.5 ^b,^*	0.4 ^b,^*	0.004
n-6	3.8	3.6 ^a^	3.9 ^b^	3.9 ^b^	0.001	3.7 *	3.4 ^a,^*	3.9 ^b^	3.7 ^c,^*	<0.001

SFAs, saturated fatty acids; MUFAs, monosaturated fatty acids; PUFAs, polyunsaturated fatty acids; n-3, omega-3 fatty acids; n-6, omega-6 fatty acids. Results are expressed as the median percentage of contribution to the total energy intake. Results are expressed as a median. Individual Usual Intakes from two 24 h dietary recalls were used. Mann-Whitney U- test was used to evaluate differences by total and age group between SRS and AMS (significant differences are marked with an asterisk [*] in median values of the AMS cohort). Kruskal–Wallis test was used to calculate differences among age groups within cohorts (significant differences are marked with superscript letters in the median values of each age group). *p*-values for this test are included in the last column. *p*-value < 0.05 was considered statistically significant.

**Table 5:** Covariance analysis (ANCOVA) of variables associated with main fatty acid intakes from two 24 h dietary recalls of the two cohorts of the Nutritional Study in the Spanish Pediatric Population (EsNuPI) (*n* = 1448).

	Spanish Reference Cohort (SRS)				Adapted Milk Consumers Cohort (AMS)			
	Total *n* = 687				Total *n* = 726			
(g)	Mean	β	CI (95%)	*p*	Mean	β	CI (95%)	*p*
**Total Fat**	61.1				56.8			
Nielsen area		−0.84	−1.25–(−0.418)	<0.001		−0.84	−1.25–(−0.418)	<0.001
Family income		0.95	−0.032–1.94	0.058		0.95	−0.032–1.94	0.058
Highest level of education achieved by one of the parents		−1.73	−3.20–(−0.25)	0.022		−1.73	−3.20–(−0.25)	0.022
**SFAs**	22.1				19.3			
Nielsen area		−0.22	−0.384–(−0.049)	0.011		−0.22	−0.384–(−0.049)	0.011
Family income		0.15	−0.25–0.54	0.47		0.15	−0.25–0.54	0.47
Highest level of education achieved by one of the parents		−0.74	−1.33–(−0.15)	0.014		−0.74	−1.33–(−0.15)	0.014
**PUFAs**	7.78				7.27			
Nielsen area		−0.17	−0.25–(−0.10)	<0.001		−0.17	−0.25–(−0.10)	<0.001
Family income		0.10	−0.084–(0.29)	0.28		0.10	−0.084–(0.29)	0.28
Highest level of education achieved by one of the parents		−0.40	−0.68–(−0.12)	0.005		−0.40	−0.68–(−0.12)	0.005
**n-3**	0.647				0.695			
Nielsen area		0.001	−0.006–0.008	0.82		0.001	−0.006–0.008	0.82
Family income		0.013	−0.003–0.030	0.109		0.013	−0.003–0.030	0.109
Highest level of education achieved by one of the parents		0.009	−0.015–0.034	0.451		0.009	−0.015–0.034	0.451
**n-6**	6.54				5.93			
Nielsen area		−0.152	−0.222–(−0.082)	<0.001		−0.152	−0.222–(−0.082)	<0.001
Family income		0.115	−0.050–0.281	0.172		0.115	−0.050–0.281	0.172
Highest level of education achieved by one of the parents		−0.387	−0.635–(−0.138)	0.002		−0.387	−0.635–(−0.138)	0.002

SFAs, saturated fatty acids; PUFAs, polyunsaturated fatty acids; n-3, omega-3 fatty acids; n-6, omega-6 fatty acids. Results are expressed as mean, standardized beta coefficient (β), confidence interval (CI) (95%), and *p*-values <0.05 were considered statistically significant.

**Table 6:** Intake of major fatty acids from two 24 h dietary recalls from two cohorts of the Nutritional Study in the Spanish Pediatric Population (EsNuPI) according to age group (*n* = 1448).

**Spanish Reference Cohort (SRS)**																	
	**Total *n* = 707**				**1 to <3 Years *n* = 162**				**3 to <6 Years *n* = 244**				**6 to <10 Years *n* = 301**				
**Fatty acids (g)**	**Mean**	**SD**	**Median**	**IQR**	**Mean**	**SD**	**Median**	**IQR**	**Mean**	**SD**	**Median**	**IQR**	**Mean**	**SD**	**Median**	**IQR**	***p***
**Myristic acid 14:0**	1.72	0.84	1.63	1.07	1.47	0.80	1.43 ^a^	1.02	1.73	0.77	1.61 ^b^	1.10	1.85	0.88	1.76 ^b^	1.03	<0.001
**Palmitic acid 16:0**	10.82	4.44	10.45	6.03	8.13	4.03	7.48 ^a^	4.52	10.96	3.74	10.66 ^b^	5.09	12.15	4.54	11.67 ^c^	6.02	<0.001
**Stearic acid 18:0**	4.17	1.95	3.96	2.26	3.05	1.60	2.89	2.04	4.23	1.65	4.14	2.01	4.72	2.10	4.33	2.42	<0.001
**Palmitoleic acid 16:1 n-7**	1.05	0.46	1.01	0.58	0.85	0.43	0.82 ^a^	0.49	1.05	0.39	1.03 ^b^	0.47	1.16	0.49	1.07 ^b^	0.63	<0.001
**Oleic acid 18:1 n-9**	22.26	8.69	21.73	10.98	16.84	8.17	16.55 ^a^	11.19	22.51	7.72	22.29 ^b^	9.81	24.97	8.39	23.83 ^c^	10.97	<0.001
**Linoleic acid 18:2 n-6**	6.48	3.57	5.85	3.94	5.03	3.28	4.45 ^a^	3.44	6.36	3.24	5.85 ^b^	3.77	7.36	3.72	6.68 ^c^	3.99	<0.001
**α-Linolenic acid 18:3 n-3**	0.46	0.19	0.44	0.21	0.36	0.15	0.35 ^a^	0.20	0.45	0.16	0.43 ^b^	0.16	0.51	0.21	0.49 ^c^	0.23	<0.001
**Arachidonic acid 20:4 n-6**	0.08	0.05	0.06	0.06	0.06	0.05	0.05 ^a^	0.05	0.08	0.05	0.07 ^b^	0.07	0.09	0.05	0.07 ^b^	0.06	<0.001
**Eicosapentaenoic acid 20:5 n-3**	0.06	0.08	0.01	0.10	0.05	0.07	0.01	0.10	0.05	0.07	0.01	0.10	0.06	0.09	0.01	0.10	0.121
**Docosapentaenoic acid 22:5 n-3**	0.05	0.05	0.03	0.04	0.03	0.03	0.02 ^a^	0.03	0.05	0.04	0.04 ^b^	0.04	0.05	0.05	0.04 ^b^	0.04	<0.001
**Docosahexaenoic acid 22:6 n-3**	0.09	0.14	0.02	0.14	0.08	0.12	0.02	0.14	0.08	0.14	0.02	0.14	0.10	0.16	0.02	0.15	0.144
**Adapted Milk Consumers Cohort (AMS)**																	
	**Total *n* = 741**				**1 to <3 Years *n* = 294**				**3 to <6 Years *n* = 262**				**6 to <10 Years *n* = 185**				
**Fatty acids (g)**	**Mean**	**SD**	**Median**	**IQR**	**Mean**	**SD**	**Median**	**IQR**	**Mean**	**SD**	**Median**	**IQR**	**Mean**	**SD**	**Median**	**IQR**	***p***
**Myristic acid 14:0**	1.12	0.71	1.02 *	0.98	0.86	0.64	0.70 ^a,^*	0.76	1.22	0.63	1.12 ^b,^*	0.93	1.38	0.77	1.29 ^b,^*	1.12	<0.001
**Palmitic acid 16:0**	8.29	4.05	7.85 *	5.28	6.16	3.67	5.52 ^a,^*	4.32	9.27	3.62	8.99 ^b,^*	4.99	10.28	3.67	9.64 ^c,^*	5.19	<0.001
**Stearic acid 18:0**	3.18	1.73	2.95	2.27	2.21	1.29	2.00 ^a,^*	1.27	3.63	1.66	3.52 ^b,^*	2.28	4.09	1.67	3.76 ^c,^*	2.05	<0.001
**Palmitoleic acid 16:1 n-7**	0.84	0.49	0.76	0.52	0.71	0.53	0.61 ^a,^*	0.46	0.92	0.42	0.84 ^b,^*	0.51	0.96	0.44	0.88 ^b,^*	0.47	<0.001
**Oleic acid 18:1 n-9**	18.60	8.30	17.74 *	11.15	14.33	7.66	13.67 ^a,^*	9.23	20.39	7.27	19.62 ^b,^*	10.29	22.85	7.54	22.64 ^c,^*	10.77	<0.001
**Linoleic acid 18:2 n-6**	5.83	3.11	5.15 *	3.82	4.48	2.38	4.15 ^a^	2.75	6.48	3.30	5.56 ^b^	4.07	7.05	3.06	6.42 ^c^	3.96	<0.001
**α-Linolenic acid 18:3 n-3**	0.44	0.20	0.42 *	0.20	0.40	0.17	0.38 ^a,^*	0.20	0.45	0.19	0.42 ^b^	0.23	0.48	0.18	0.45 ^b,^*	0.19	<0.001
**Arachidonic acid 20:4 n-6**	0.07	0.05	0.05 *	0.07	0.06	0.05	0.04 ^a^	0.07	0.07	0.05	0.05 ^b,^*	0.07	0.08	0.05	0.06 ^b,^*	0.07	<0.001
**Eicosapentaenoic acid 20:5 n-3**	0.08	0.10	0.06 *	0.13	0.05	0.07	0.01 ^a^	0.09	0.09	0.10	0.07 ^b,^*	0.14	0.13	0.11	0.11 ^c,^*	0.13	<0.001
**Docosapentaenoic acid 22:5 n-3**	0.04	0.04	0.03 *	0.04	0.03	0.04	0.02 ^a^	0.03	0.04	0.04	0.03 ^b,^*	0.04	0.05	0.05	0.04 ^c^	0.04	<0.001
**Docosahexaenoic acid 22:6 n-3**	0.13	0.16	0.09 *	0.14	0.13	0.15	0.10 *	0.14	0.13	0.15	0.09 *	0.15	0.14	0.18	0.08 *	0.12	0.903

Average gram intake values from two 24 h dietary recalls were used. Results are expressed as mean, standard deviation (SD), median, and interquartile range (IQR). Mann–Whitney U-test was used to evaluate differences by total and age group between SRS and AMS (significant differences are marked with an asterisk * in median values of the AMS cohort). Kruskal–Wallis test was used to calculate differences among age groups within cohorts (significant differences are marked with superscript letters in the median values of each age group). *p*-values for this test are included in the last column. *p*-value <0.05 was considered statistically significant.

**Table 7:** Percentages of children meeting and not meeting European Food Safe Authority (EFSA) recommendations for main fatty acids by cohort and age group Figure 1448 ¥.

**Spanish Reference Cohort (SRS)**																	
	**Total**				**1 to <3 Years**				**3 to <6 Years**				**6 to <10 Years**				
	**n**	**% BR**	**% MR**	**% AR£**	**n**	**% BR**	**% MR**	**% AR£**	**n**	**% BR**	**% MR**	**% AR£**	**n**	**% BR**	**% MR**	**% AR£**	***p***
**Total Fat**	707	13.7	41.7	44.6	162	42.0	35.8 ^a^	22.2	244	11.9	50.8 ^b^	37.3	301	0.0	37.5 ^a^	62.5	<0.001
**Linoleic acid**	707	57.4	26.4	16.1	162	66.7	25.3	8.0	244	56.6	27.5	16.0	301	53.2	26.2	20.6	NS
**α-Linolenic acid**	707	99.0	1.0	0.0	162	98.8	1.2	0.0	244	99.6	0.4	0.0	301	98.7	1.3	0.0	NS
**EPA+DHA**	707	73.4	2.4	24.2	162	73.5	1.9	24.7	244	75.0	2.0	23.0	301	72.1	3.0	24.9	NS
**Adapted Milk Consumers Cohort (AMS)**																	
	**Total**				**1 to <3 Years**				**3 to <6 Years**				**6 to <10 Years**				
	**n**	**% BR**	**% MR**	**% AR£**	**n**	**% BR**	**% MR**	**% AR£**	**n**	**% BR**	**% MR**	**% AR£**	**n**	**% BR**	**% MR**	**% AR£**	***p***
**Total Fat**	741	27.9 *	37.1 *	35.0 *	294	56.5 *	35.0 ^a^	8.5 *	262	15.6	45.0 ^b^	39.3	185	0.0	29.2 ^a^	70.8	<0.001
**Linoleic acid**	741	64.6 *	24.4 *	10.9 *	294	77.9 *	13.9 ^a,^*	8.2	262	53.8	32.4 ^b^	13.7	185	58.9	29.7 ^b^	11.4 *	<0.001
**α-Linolenic acid**	741	96.6 *	2.4 *	0.9 *	294	93.2 *	4.8 ^a^	2.0	262	99.2	0.4 ^b^	0.4	185	98.4	1.6 ^a,b^	0.0	<0.001
**EPA+DHA**	741	59.2 *	2.6 *	38.2 *	294	56.1 *	4.4	39.5 *	262	63.4 *	1.1	35.5 *	185	58.4 *	1.6	40.0 *	NS

The percentage for inadequacy was calculated by comparing with EFSA recommendations. First column: percentage below recommendations (BR); second column: percentage meeting recommendations MR); third column: percentage above recommendations (AR). DHA, docosahexaenoic acid, 22:6 n-3; EPA, eicosapentaenoic acid, 20:5 n-3; NS, no significance. Results are expressed in percentage (%). ¥ Individual usual intake for two 24 h dietary recalls was used for total fats, linoleic acid, α-linolenic acid, SFAs, and PUFAs. Average gram intake values from two 24 h dietary recalls were used for EPA+DHA. Chi-square test was used to evaluate differences by total and age group between SRS and AMS (significant differences are marked with an asterisk * in the percentage values of the AMS cohort). Chi-square test analysis was used to calculate differences among age groups within cohorts (significant differences are marked with superscript letters in the value of each age group that is meeting the recommendations). **£** The percentages of children above the recommendations do not exceed the tolerable upper intake limit. *p*-values for this test are included in the last column of the table. *p*-value <0.05 was considered statistically significant.

**Table 8:** Percentages of children meeting and not meeting Food and Agriculture Organization United Nations (UN-FAO) recommendations for main fatty acids by cohort and age categories for Nutritional Study in the Spanish Pediatric Population (EsNuPI) (*n* = 1448‡) ¥.

**Spanish Reference Cohort (SRS)**																	
	**Total**				**1 to <3 Years**				**3 to <6 Years**				**6 to <10 Years**				
	**n**	**% BR**	**% MR**	**% AR£**	**n**	**% BR**	**% MR**	**% AR£**	**n**	**% BR**	**% MR**	**% AR£**	**n**	**% BR**	**% MR**	**% AR£**	***p***
**Total Fat**	707	4.0	38.2	57.9	162	16.0	30.9 ^a^	53.1	244	0.8	43.9 ^b^	55.3	301	0.0	37.5 ^a,b^	62.5	<0.001
**SFAs ‡**	629	0.6	1.1	98.3	84	2.4	3.6	94.0	244	0.4	0.8	98.8	301	0.3	0.7	99.0	NS
**PUFAs**	707	88.8	11.2	0.0	162	51.9	48.1 ^a^	0.0	244	100.0	0.0 ^b^	0.0	301	99.7	0.3 ^b^	0.0	<0.001
**EPA+DHA**	707	67.8	5.0	27.3	162	66.7	4.9	28.4	244	66.0	4.5	29.5	301	69.8	5.3	24.9	NS
**Adapted Milk Consumers Cohort (AMS)**																	
	**Total**				**1 to <3 Years**				**3 to <6 Years**				**6 to <10 Years**				
	**n**	**% BR**	**% MR**	**% AR£**	**n**	**% BR**	**% MR**	**% AR£**	**n**	**% BR**	**% MR**	**% AR£**	**n**	**% BR**	**% MR**	**% AR£**	***p***
**Total Fat**	741	11.6 *	35.6 *	52.8 *	294	29.3 *	33.7 ^a,b^	37.1 *	262	0.0	42.4 ^b^	57.6	185	0.0	29.2 ^a^	70.8	<0.001
**SFAs ‡**	582	0.5	2.7	96.7	135	2.2	7.4 ^a^	90.4	262	0.0	1.5 ^b^	98.5	185	0.0	1.1 ^b^	98.9	<0.001
**PUFAs**	741	78.5 *	21.5 *	0.0 *	294	45.9	54.1 ^a^	0.0	262	100	0.0 ^b^	0.0	185	100	0.0 ^b^	0.0	<0.001
**EPA+DHA**	741	46.0 *	12.0 *	42.0 *	294	47.3 *	13.3 *	39.5 *	262	42.4 *	11.5 *	46.2 *	185	49.2 *	10.8 *	40.0 *	NS

The percentage for inadequacy was calculated by comparing with UN-FAO recommendations. First column: percentage below recommendations (BR); second column: percentage meeting recommendations (MR); third column: percentage above recommendations (AR). DHA, docosahexaenoic acid 22:6 n-3; EPA, eicosapentaenoic acid 20:5 n-3; SFAs, saturated fatty acids; PUFAs, polyunsaturated fatty acids; NS, no significance. Results expressed in percentage (%). ¥ Individual usual intake for two 24 h dietary recalls was used to calculate the presented table for total fats, linoleic acid, α-linolenic acid, SFAs, and PUFAs. Average gram intake values from two 24 h dietary recalls were used for EPA+DHA. Chi-square test was used to evaluate differences by total and age group between SRS and AMS (significant differences are marked with an asterisk [*] in percentage values of the AMS cohort). Chi-square test analysis was used to calculate differences among age groups within cohorts (significant differences are marked with superscript letters in the value of each age group that are meeting the recommendations) *p*-values for this test are included in the last column. *p*-value <0.05 was considered statistically significant. **£** The percentages of children above the recommendations do not exceed the tolerable upper intake limit. ‡ Recommendations for SFAs intake are available only for children aged 2 to <10 years; therefore, children <2 years were excluded from these analyses (*n* = 1211)**.**

**Table 9:** Odds ratios (OR) and 95% confidence intervals (CI) for intake equal to or higher than the median of fat, saturated fatty acids, and monounsaturated fatty acids relative to family and personal factors in the reference cohort of the Nutritional Study in the Spanish Pediatric Population (EsNuPI) (*n* = 707).

Spanish Reference Cohort (SRS)										
		Total Fat (g/Day) (≥P50) ^†^			SFAs (g/Day) (≥P50) ^†^			MUFAs (g/Day) (≥P50) ^†^		
Factor	Subcategories	OR	CI	*p*	OR	CI	*p*	OR	CI	*p*
Sex	Boys	1			1					
	Girls	1.183	0.845–1.656	0.328	1.046	0.753–1.454	0.787	1.424	1.017–1.994	0.039 *
Age	1 to <3 years	1			1					
	3 to <6 years	0.089	0.054–0.146	<0.001 *	0.150	0.095–0.237	<0.001 *	0.081	0.049–0.135	<0.001 *
	6 to <10 years	0.416	0.290–0.597	<0.001 *	0.580	0.406–0.830	0.003 *	0.414	0.288–0.596	<0.001 *
Number of feeding bottles or glasses of milk per day	Less than 2	1			1			1		
	2 or more	0.894	0.626–1.277	0.539	0.758	0.535–1.074	0.119	1.032	0.721–1.477	0.864
Physical activity level (PAL)	≥P50 by sex and age	0.756	0.371–1.540	0.441	0.917	0.460–1.831	0.807	0.889	0.434–1.821	0.748
Size of municipality (n)	50,000–300,000	1			1			1		
	>300,000	0.809	0.577–1.132	0.216	0.790	0.570–1.095	0.156	1.063	0.757–1.491	0.725
Family income (€)	≤1500	1			1					
	1501–2000	1.095	0.660–1.817	0.724	1.196	0.735–1.946	0.470	1.071	0.645–1.780	0.790
	≥2000	1.258	0.748–2.115	0.397	0.983	0.594–1.627	0.948	1.134	0.674–1.909	0.635
	Not known/no answer	1.251	0.801–1.954	0.325	1.280	0.830–1.973	0.264	1.151	0.736–1.799	0.537
Highest level of education achieved by one parent	≤10 years of education	1			1			1		
	Secondary education	1.074	0.676–1.705	0.763	1.621	1.037–2.535	0.034 *	0.813	0.512–1.292	0.381
	University studies	0.911	0.621–1.337	0.633	1.050	0.726–1.517	0.796	0.814	0.554–1.196	0.294
Anthropometry	z-height for age	1.085	0.979–1.202	0.121	1.067	0.967–1.178	0.197	1.064	0.960–1.180	0.236
	z-BMI for age	1.076	0.973–1.191	0.152	1.063	0.963–1.172	0.225	1.029	0.931–1.137	0.581

SFAs, saturated fatty acids; MUFAs, monounsaturated fatty acids; PUFAs, polyunsaturated fatty acids; n-3: omega-3 fatty acids; n-6: omega-6 fatty acids; OR, odds ratio; CI, confidence intervals; PAL, physical activity level; BMI, body mass index. ^†^ P50 or median was calculated in the reference cohort for each nutrient by sex and age group and used to categorize children according to whether their usual nutrient intake was below or above the median. z-BMI/age and z-height/age were defined according to World Health Organization international growth patterns. * *p*-value < 0.05 was considered statistically significant.

**Table 10:** Odds ratios and 95% confidence intervals for intake equal to or higher than the median of polyunsaturated fatty acids, n-3 fatty acids, n-6 fatty acids and, n-6:n-3 ratio relative to family and personal factors in the reference cohort of the Nutritional Study in the Spanish Pediatric Population (EsNuPI) (*n* = 707).

Spanish Reference Cohort (SRS)													
		PUFAs (g/Day) (≥P50) ^†^			n-3 (g/Day) (≥P50) ^†^			n-6 (g/Day) (≥P50) ^†^			n-6:n-3 (g/Day) (≥P50) ^†^		
Factor	Subcategories	OR	CI	*p*	OR	CI	*p*	OR	CI	*p*	OR	CI	*p*
Sex	Boys	1			1			1			1		
	Girls	1.386	0.984–1.951	0.062	1.043	0.758–1.435	0.797	1.144	0.832–1.571	0.408	1.194	0.876–1.627	0.262
Age	1 to <3 years	1			1			1			1		
	3 to <6 years	0.087	0.052–0.145	<0.001 *	0.323	0.213–0.489	<0.001 *	0.293	0.191–0.449	<0.001 *	0.601	0.403–0.896	0.013 *
	6 to <10 years	0.443	0.306–0.642	<0.001 *	0.659	0.462–0.939	0.021 *	0.697	0.488–0.996	0.048 *	0.794	0.560–1.125	0.195
Number of feeding bottles or glasses of milk per day	Less than 2	1			1			1			1		
	2 or more	1.283	0.896–1.837	0.173	0.898	0.641–1.260	0.535	1.087	0.773–1.528	0.632	1.299	0.933–1.808	0.122
Physical activity level (PAL)	≥P50 by sex and age	0.459	0.224–0.942	0.034 *	0.789	0.415–1.500	0.470	0.795	0.413–1.530	0.492	1.464	0.782–2.740	0.233
Size of municipality (n)	50,000–300,000	1			1			1			1		
	>300,000	0.719	0.512–1.009	0.057	0.817	0.597–1.120	0.210	0.745	0.543–1.023	0.069	0.869	0.637–1.184	0.374
Family income (€)	≤1500				1			1			1		
	1501–2000	0.979	0.588–1.628	0.934	0.775	0.483–1.243	0.290	1.253	0.778–2.020	0.353	1.119	0.703–1.780	0.636
	≥2000	1.168	0.693–1.969	0.559	0.755	0.464–1.229	0.258	1.271	0.779–2.074	0.337	1.356	0.840–2.187	0.212
	Not known/no answer	1.077	0.688–1.684	0.746	0.882	0.581–1.341	0.558	1.093	0.717–1.666	0.679	1.134	0.751–1.712	0.549
Highest level of education achieved by one parent	≤10 years of education	1			1			1			1		
	Secondary education	1.310	0.822–2.087	0.256	0.951	0.619–1.462	0.818	1.583	1.026–2.441	0.038 *	1.353	0.887–2.065	0.160
	University studies	0.801	0.545–1.178	0.260	0.838	0.585–1.201	0.335	1.116	0.778–1.600	0.552	1.118	0.785–1.593	0.536
Anthropometry	z-height for age	1.094	0.986–1.214	0.091	0.966	0.876–1.065	0.484	1.085	0.986–1.194	0.096	1.043	0.947–1.149	0.395
	z-BMI for age	1.007	0.909–1.114	0.898	1.115	1.011–1.229	0.029 *	1.025	0.933–1.127	0.604	0.946	0.863–1.037	0.237

SFAs, saturated fatty acids; MUFAs, monounsaturated fatty acids; PUFAs, polyunsaturated fatty acids; n-3: omega-3 fatty acids; n-6: omega-6 fatty acids; OR, odds ratio; CI, confidence intervals; PAL, physical activity level; BMI, body mass index. ^†^ P50 or median was calculated in the reference cohort for each nutrient by sex and age group and used to categorize children according to whether their usual nutrient intake was below or above the median. z-BMI/age and z-height/age were defined according to World Health Organization international growth patterns. * *p*-value < 0.05 was considered statistically significant.

**Table 11:** Odds ratios and 95% confidence intervals for intake equal to or higher than the median of fat, saturated fatty acids, and monounsaturated fatty acids relative to family and personal factors in adapted milk consumers cohort of Nutritional Study in the Spanish Pediatric Population (EsNuPI) (*n* = 741).

Adapted Milk Consumers Cohort (AMS)										
		Total Fat (g/Day) (≥P50) ^†^			SFAs (g/Day) (≥P50) ^†^			MUFAs (g/Day) (≥P50) ^†^		
Factor	Subcategories	OR	CI	*p*	OR	CI	*p*	OR	CI	*p*
Sex	Boys	1			1			1		
	Girls	0.796	0.541–1.172	0.247	0.905	0.622–1.317	0.602	0.812	0.559–1.181	0.276
Age	1 to <3 years	1			1			1		
	3 to <6 years	0.011	0.005–0.021	<0.001 *	0.021	0.012–0.037	<0.001 *	0.018	0.010–0.033	<0.001 *
	6 to <10 years	0.104	0.055–0.197	<0.001 *	0.199	0.116–0.341	<0.001 *	0.149	0.084–0.264	<0.001 *
Number of feeding bottles or glasses of milk per day	Less than 2	1			1			1		
	2 or more	1.232	0.784–1.936	0.365	1.146	0.734–1.790	0.549	1.162	0.746–1.810	0.508
Physical activity level (PAL)	≥P50 by sex and age	0.704	0.319-1.551	0.384	0.651	0.303–1.399	0.271	0.754	0.349–1.628	0.472
Size of municipality (n)	50,000–300,000	1			1			1		
	>300,000	1.198	0.802–1.789	0.377	0.788	0.542–1.147	0.214	1.264	0.862–1.855	0.230
Family income (€)	≤1500	1						1		
	1501–2000	0.659	0.381–1.142	0.137	0.825	0.480–1.417	0.485	0.751	0.442–1.278	0.292
	≥2000	0.487	0.271–0.873	0.016 *	0.622	0.354–1.093	0.099	0.523	0.297–0.920	0.025 *
	Not known/no answer	0.763	0.467–1.246	0.280	0.810	0.500-1.312	0.392	0.807	0.502–1.298	0.377
Highest level of education achieved by one parent	≤10 years of education	1			1			1		
	Secondary education	1.390	0.771–2.506	0.273	1.524	0.863–2.691	0.146	1.332	0.756–2.348	0.321
	University studies	1.236	0.806–1.894	0.331	1.159	0.766–1.752	0.485	1.273	0.842–1.925	0.252
Anthropometry	z- height for age	1.079	0.966–1.205	0.179	1.098	0.987–1.223	0.086	1.102	0.990–1.226	0.075
	z- BMI for age	1.016	0.895–1.152	0.810	1.071	0.950–1.208	0.259	1.028	0.910–1.161	0.662

SFAs, saturated fatty acids; MUFAs, monounsaturated fatty acids; PUFAs, polyunsaturated fatty acids; n-3, omega-3 fatty acids; n-6, omega-6 fatty acids; OR, odds ratio; CI, confidence intervals; PAL, physical activity level; BMI, body mass index. ^†^ P50 or median was calculated for adapted milk consumers cohort for each nutrient by sex and age group and used to categorize children according to whether their usual nutrient intake was below or above the median. z-BMI/age and z-height/age were defined according to World Health Organization (WHO) international growth patterns. * *p*-value < 0.05 was considered statistically significant.

**Table 12:** Odds ratios and 95% confidence intervals for intake equal to or higher than the median of n-3 fatty acids, n-6 fatty acids, and n-6:n-3 ratio relative to family and personal factors in adapted milk consumers cohort of Nutritional Study in the Spanish Pediatric Population (EsNuPI) (*n* = 741).

Adapted Milk Consumers Cohort (AMS)													
		PUFAs (g/Day) (≥P50) ^†^			n-3 (g/Day) (≥P50) ^†^			n-6 (g/Day) (≥P50) ^†^			n-6:n-3 (g/Day) (≥P50) ^†^		
Factor	Subcategories	OR	CI	*p*	OR	CI	*p*	OR	CI	*p*	OR	CI	*p*
Sex	Boys	1			1			1			1		
	Girls	1.023	0.684–1.530	0.912	0.918	0.679–1.242	0.581	0.975	0.714–1.331	0.872	1.041	0.770–1.406	0.796
Age	1 to <3 years	1			1			1			1		
	3 to <6 years	0.009	0.004–0.018	<0.001 *	0.365	0.247–0.540	<0.001 *	0.186	0.124–0.280	<0.001 *	0.432	0.295–0.632	<0.001 *
	6 to <10 years	0.098	0.049–0.196	<0.001 *	0.697	0.469–1.034	0.073	0.530	0.353–0.795	0.002 *	1.008	0.685–1.483	0.968
Number of feeding bottles or glasses of milk per day	Less than 2	1			1			1			1		
	2 or more	1.552	0.974–2.474	0.065	1.113	0.777–1.595	0.559	0.962	0.662–1.398	0.839	0.930	0.650–1.331	0.692
Physical activity level (PAL)	≥P50 by sex and age	1.037	0.448–2.400	0.932	0.882	0.480–1.621	0.687	1.007	0.533–1.905	0.982	1.106	0.601–2.039	0.745
Size of municipality (n)	50,000–300,000	1			1			1			1		
	>300,000	1.117	0.738–1.692	0.600	0.985	0.719–1.347	0.922	1.185	0.863–1.627	0.295	0.991	0.725–1.354	0.955
Family income (€)	≤1500	1			1			1			1		
	1501–2000	1.348	0.760–2.390	0.306	0.639	0.407–1.003	0.051	0.710	0.455–1.107	0.131	1.077	0.687–1.688	0.747
	≥2000	1.593	0.323–1.087	0.091	0.567	0.358–0.898	0.016 *	0.696	0.436–1.110	0.128	0.770	0.489–1.215	0.262
	Not known/no answer	0.732	0.439–1.218	0.230	0.940	0.636–1.387	0.754	0.673	0.450–1.006	0.053	0.821	0.557–1.209	0.318
Highest level of education achieved by one parent	≤10 years of education	1			1			1			1		
	Secondary education	1.416	0.768–2.610	0.266	0.870	0.552–1.371	0.547	1.412	0.883–2.256	0.149	1.562	1.021–2.390	0.040 *
	University studies	1.233	0.791–1.922	0.355	1.327	0.948–1.857	0.099	1.213	0.858–1.715	0.274	1.066	0.767–1.483	0.703
Anthropometry	z-height for age	1.185	1.052–1.335	0.005 *	1.019	0.937–1.109	0.657	1.017	0.932–1.109	0.712	1.031	0.949–1.121	0.467
	z-BMI for age	1.089	0.955–1.243	0.204	0.989	0.898–1.089	0.819	0.998	0.904–1.102	0.967	0.985	0.895–1.084	0.756

SFAs, saturated fatty acids; MUFAs, monounsaturated fatty acids; PUFAs, polyunsaturated fatty acids; n-3, omega-3 fatty acids; n-6, omega-6 fatty acids; OR, odds ratio; CI, confidence intervals; PAL, physical activity level; BMI, body mass index. ^†^ P50 or median was calculated for adapted milk consumers cohort for each nutrient by sex and age group and used to categorize children according to whether their usual nutrient intake was below or above the median. z-BMI/age and z-height/age were defined according to World Health Organization (WHO) international growth patterns. * *p*-value < 0.05 was considered statistically significant.

## Supplementary Materials

The following are available online at https://www.mdpi.com/2072-6643/12/8/2467/s1, Figure S1: Contribution (%) of the 18 food groups to monounsaturated fatty acids (MUFAs) intake in the EsNuPI study population (Spanish Reference Cohort (SRS) and Adapted Milk Consumers Cohort (AMS)) according to age group (Gp 1, 1 to <3 years; Gp 2, 3 to <6 years; Gp 3, 6 to <10 years), Figure S2: Contribution (%) of the 18 food groups to polyunsaturated fatty acids (PUFAs) intake in the EsNuPI study population (Spanish Reference Cohort (SRS) and Adapted Milk Consumers Cohort (AMS)) according to age group (Gp 1, 1 to <3 years; Gp 2, 3 to <6 years; Gp 3, 6 to <10 years), Figure S3: Contribution (%) of the 18 food groups to n-6 intake in the EsNuPI study population (Spanish Reference Cohort (SRS) and Adapted Milk Consumers Cohort (AMS)) according to age group (Gp 1, 1 to <3 years; Gp 2, 3 to <6 years; Gp 3, 6 to <10 years), Figure S4: Contribution (%) of the 18 food groups to n-3 intake in the EsNuPI study population (Spanish Reference Cohort (SRS) and Adapted Milk Consumers Cohort (AMS)) according to age group (Gp 1, 1 to <3 years; Gp 2, 3 to <6 years; Gp 3, 6 to <10 years), Figure S5: Contribution (%) of the 18 food group to eicosapentaenoic acid (EPA) intake in the EsNuPI study population (Spanish Reference Cohort (SRS) and Adapted Milk Consumers Cohort (AMS)) according to age group (Gp 1, 1 to <3 years; Gp 2, 3 to <6 years; Gp 3, 6 to <10 years, Table S1: Total lipids and main fatty acids intake from two 24 h dietary recalls from two cohorts of Nutritional Study in Spanish Pediatric Population (EsNuPI): Spanish reference cohort and adapted milk consumer cohort, according to age group and sex (n = 1448), Table S2: Total lipid and main fatty acid intake based on two 24 h dietary recalls from plausible reporters of two cohorts of Nutritional Study in Spanish Pediatric Population (EsNuPI), according to age group (n = 1216), Table S3: Intake of major fatty acids based on two 24 h dietary recalls from two cohorts of Nutritional Study in Spanish Pediatric Population (EsNuPI), according to age group and sex (n = 1448), Table S4: Percentages of children meeting and not meeting European Food Safe Authority (EFSA) recommendations for main fatty acids by cohort and age group among plausible reporters of the Nutritional Study in Spanish Pediatric Population (EsNuPI) (n =1216) ¥, Table S5: Percentages of children meeting and not meeting Food and Agriculture Organization (FAO) recommendations for main fatty acids by cohort and age group among plausible reporters of the Nutritional Study in Spanish Pediatric Population (EsNuPI) (n = 1216‡) ¥.
