# Supplementary material for: Dietary Intake, Nutritional Adequacy and Food Sources of Total Fat and Fatty Acids, and Relationships with Personal and Family Factors in Spanish Children Aged One to <10 Years: Results of the EsNuPI Study
Source: Nutrients. 2020 Aug 16;12(8):2467. doi: 10.3390/nu12082467 (PMC7468841; doi:10.3390/nu12082467)
Supplement: Supplementary file 1 [file nutrients-12-02467-s001.pdf]

Supplementary Table 1. Total lipids and main fatty acids intake from two 24 h dietary recalls from two cohorts of Nutritional Study in Spanish Pediatric Population (EsNuPI): Spanish reference cohort and adapted milk consumer cohort, according to age group and sex (n = 1448).

Spanish Reference Cohort (SRS)												
	Total			1 to <3 years			3 to <6 years			6 to <10 years		
	Total	Boys	Girls	Total	Boys	Girls	Total	Boys	Girls	Total	Boys	Girls
(g) *n*	707	357	350	162	84	78	244	122	122	301	151	150
**Total Fat**	59.77	60.18	58.79	47.34	48.87	46.95	60.34	60.13	61.18	64.77	65.02	64.77
	(24.86)	(25.57)	(26.29)	(22.89)	(22.72)	(23.53)	(20.73)	(20.14)	(21.51)	(27.29)	(27.27)	(28.93)
**SFAs**	21.23	21.35	20.93	16.88	16.44	17.50	22.18	21.62	22.76	23.45	23.96	22.93
	(10.75)	(10.70)	(11.22)	(8.53)	(8.86)	(8.16)	(9.44)	(8.63)	(9.89)	(10.60)	(11.02)	(11.35)
**MUFAs**	25.13	25.50	24.5	19.25	19.37	18.63	25.29	25.29	25.42	27.62	28.44	26.63
	(11.38)	(11.16)	(11.49)	(11.56)	(11.96)	(11.09)	(10.33)	(10.61)	(10.91)	(11.83)	(12.57)	(12.05)
**PUFAs**	7.16	7.20	6.94	5.15	5.30	4.78	7.16	7.15	7.17	8.17	8.38	8.04
	(4.56)	(4.22)	(4.74)	(4.16)	(4.55)	(3.76)	(3.83)	(3.88)	(3.94)	(4.21)	(4.09)	(4.43)
**n-3**	0.59	0.59	0.60	0.49	0.52	0.44	0.59	0.58	0.61	0.66	0.66	0.66
	(0.35)	(0.36)	(0.35)	(0.30)	(0.32)	(0.28)	(0.33)	(0.35)	(0.35)	(0.37)	(0.34)	(0.38)
**n-6**	5.91	6.05	5.82	4.51	4.75	4.25	5.91	5.98	5.81	6.75	6.93	6.55
	(3.99)	(3.78)	(4.02)	(3.51)	(3.61)	(3.13)	(3.77)	(3.65)	(3.78)	(4.01)	(3.83)	(4.26)
**n-6: n-3**	10.14	10.22	9.92	9.25	8.25	9.61	10.13	10.42	9.53	10.50	10.68	10.44
	(6.09)	(6.27)	(6.24)	(6.36)	(5.89)	(7.66)	(5.95)	(6.04)	(5.83)	(5.81)	(5.73)	(5.71)

Adapted Milk Consumers Cohort (AMS)												
	Total			1 to <3 years			3 to <6 years			6 to <10 years		
	Total	Boys	Girls	Total	Boys	Girls	Total	Boys	Girls	Total	Boys	Girls
(g) *n*	741	371	370	294	144	150	262	128	134	185	99	86
**Total Fat**	54.94*	54.14*	56.11*	43.20	43.54	43.14	61.12	57.02	62.61	64.90	65.44	64.10
	(27.42)	(28.15)	(27.17)	(20.71)	(21.14)	(20.73)	(26.85)	(27.06)	(26.12)	(24.44)	(25.79)	(22.76)
**SFAs**	18.46*	18.23*	18.77*	14.90	15.22	14.49*	20.88	20.17	21.15	22.00	22.17	21.65
	(10.48)	(10.38)	(10.52)	(7.91)	(7.63)	(8.12)	(10.57)	(11.06)	(10.22)	(9.75)	(9.80)	(9.89)
**MUFAs**	23.56*	23.66*	23.37	18.38	18.45	18.28	25.40	24.40	25.73	28.00	27.55	28.33
	(12.14)	(12.77)	(12.06)	(11.12)	(11.55)	(10.42)	(12.10)	(11.96)	(12.31)	(11.26)	(10.59)	(11.20)
**PUFAs**	6.60*	6.60*	6.60	4.90	4.88	4.91	7.50	7.45	7.56	8.51	8.72	8.34
	(4.36)	(4.54)	(4.41)	(3.57)	(3.74)	(3.48)	(4.67)	(4.32)	(4.81)	(4.38)	(4.72)	(3.66)
**n-3**	0.64*	0.63*	0.64*	0.58	0.58	0.58*	0.68	0.68*	0.68*	0.71	0.71	0.72
	(0.34)	(0.35)	(0.34)	(0.29)	(0.28)	(0.32)	(0.36)	(0.37)	(0.36)	(0.37)	(0.36)	(0.36)

**n-6**	5.21* (3.82)	5.23* (3.98)	5.18* (3.75)	4.20 (2.75)	4.15 (2.87)	4.23 (2.64)	5.62 (4.08)	5.61 (3.72)	5.64 (4.30)	6.53 (3.99)	6.61 (4.42)	6.48 (3.59)
**n-6: n-3**	8.38* (5.29)	8.51* (5.27)	8.30* (5.32)	7.33 (4.54)	7.24* (4.52)	7.42* (4.66)	9.21 (5.33)	9.51* (5.70)	9.02 (4.90)	8.91 (4.75)	8.94* (4.99)	8.89 (4.59)

SFAs: saturated fatty acids; MUFAs, monounsaturated fatty acids; PUFAs, polyunsaturated fatty acids; n-3, omega-3 fatty acids; n-6, omega-6 fatty acids. Average gram intake values for two 24 h dietary recalls were used. Results are expressed as median ± interquartile range (in brackets). Mann–Whitney U test was used to evaluate differences by total and sex groups between SRS and AMS (significant differences are marked with an asterisk [*] in the median values of the AMS). p-value < 0.05 was considered statistically significant.

Supplementary Table 2. Total lipid and main fatty acid intake based on two 24 h dietary recalls from plausible reporters of two cohorts of Nutritional Study in Spanish Pediatric Population (EsNuPI), according to age group (n = 1216).

**Spanish Reference Cohort (SRS)**																	
	**Total** n = 598				**1 to <3 years** n = 120				**3 to <6 years** n = 211				**6 to <10 years** n = 267				
**(g)**	**Mean**	**SD**	**Median**	**IQR**	**Mean**	**SD**	**Median**	**IQR**	**Mean**	**SD**	**Median**	**IQR**	**Mean**	**SD**	**Median**	**IQR**	p
**Total Fat**	61.43	18.97	60.13	23.46	47.25	14.80	46.35^{a}	19.61	60.90	15.88	60.15^{b}	19.96	68.23	19.32	65.51^{c}	25.47	<0.001
**SFAs**	22.24	8.17	21.12	10.33	16.38	7.21	15.95^{a}	7.09	22.42	6.67	22.05^{b}	8.82	24.74	8.35	23.81^{c}	10.42	<0.001
**MUFA**	25.44	8.93	25.24	10.97	18.14	7.95	18.68^{a}	9.68	25.61	7.44	25.50^{b}	10.07	28.59	8.53	27.97^{c}	10.74	<0.001
**PUFA**	7.80	3.70	7.23	4.39	5.30	3.07	5.07^{a}	4.27	7.73	3.23	7.15^{b}	3.40	8.98	3.74	8.30^{c}	4.10	<0.001
**n-3**	0.64	0.29	0.60	0.35	0.52	0.28	0.45^{a}	0.29	0.63	0.26	0.59^{b}	0.31	0.70	0.29	0.66^{c}	0.35	<0.001
**n-6**	6.56	3.36	6.03	3.80	4.80	2.50	4.38^{a}	3.39	6.45	3.11	5.90^{b}	3.35	7.44	3.57	6.91^{c}	3.98	<0.001
**n-6: n-3**	11.54	7.38	10.22	6.18	11.92	11.73	8.59^{a}	6.87	11.21	5.32	10.19	6.39	11.63	6.19	10.56^{b}	5.78	0.030

**Adapted Milk Consumers Cohort (AMS)**																	
	**Total** n = 618				**1 to <3 years** n = 236				**3 to <6 years** n = 224				**6 to <10 years** n = 158				
**(g)**	**Mean**	**SD**	**Median**	**IQR**	**Mean**	**SD**	**Median**	**IQR**	**Mean**	**SD**	**Median**	**IQR**	**Mean**	**SD**	**Median**	**IQR**	p
**Total Fat**	56.19	18.23	54.16*	25.60	44.39	12.65	42.65^{a}	17.18	59.89	16.75	58.60^{b}	24.89	68.60	16.87	65.47^{c}	24.02	<0.001
**SFAs**	19.22	7.24	18.37*	9.85	14.71	5.00	14.45^{a*}	6.87	20.70	6.57	20.31^{b*}	9.41	23.85	7.20	22.19^{c}	9.95	<0.001
**MUFA**	23.61	8.81	23.44*	11.92	17.89	7.03	17.87^{a}	10.48	25.55	7.64	25.12^{b}	10.92	29.41	7.74	28.84^{c}	10.43	<0.001
**PUFA**	7.19	3.50	6.59*	4.37	5.02	2.59	4.77^{a}	3.36	7.99	3.23	7.45^{b}	4.36	9.30	3.30	8.58^{c}	4.17	<0.001
**n-3**	0.69	0.32	0.64*	0.33	0.59	0.25	0.56^{a*}	0.28	0.70	0.31	0.65^{b*}	0.33	0.82	0.38	0.73^{c*}	0.37	<0.001
**n-6**	5.85	3.05	5.21*	3.68	4.42	2.29	4.15^{a}	2.57	6.37	3.15	5.51^{b}	3.80	7.26	3.04	6.68^{c}	3.93	<0.001
**n-6: n-3**	9.22	5.14	8.41*	5.43	8.16	5.79	7.19^{a*}	4.63	9.89	4.66	9.42^{b*}	5.39	9.84	4.49	9.01^{b*}	4.92	<0.001

SFAs: saturated fatty acids; MUFAs, monounsaturated fatty acids; PUFAs, polyunsaturated fatty acids; n-3, omega-3 fatty acids; n-6, omega-6 fatty acids. Average gram intake values from two 24 h dietary recalls were used. Results are expressed as mean, standard deviation (SD), median, and interquartile range (IQR). Mann–Whitney U test was used to evaluate differences by total and age groups between SRS and AMS (significant differences are marked with an asterisk [*] in the median values of the AMS cohort). Kruskal–Wallis test was used to calculate differences among age groups within cohorts (significant differences are marked with superscript letters in median values of each age group). p-values for this test are included in the last column. p-value < 0.05 was considered statistically significant.

Supplementary Table 3. Intake of major fatty acids based on two 24 h dietary recalls from two cohorts of Nutritional Study in Spanish Pediatric Population (EsNuPI), according to age group and sex (n = 1448).

**Spanish Reference Cohort (SRS)**												
	**Total**			**1 to <3 years**			**3 to <6 years**			**6 to <10 years**		
	**Total**	**Boys**	**Girls**	**Total**	**Boys**	**Girls**	**Total**	**Boys**	**Girls**	**Total**	**Boys**	**Girls**
**Fatty acids (g)** ***n***	707	357	350	162	84	78	244	122	122	301	151	150
**Myristic acid 14:0**	1.63	1.62	1.64	1.43	1.40	1.43	1.61	1.55	1.71	1.76	1.84	1.68
	(1.07)	(1.06)	(1.08)	(1.02)	(1.20)	(0.91)	(1.10)	(1.01)	(1.06)	(1.03)	(1.00)	(1.08)
**Palmitic acid 16:0**	10.45	10.53	10.40	7.48	7.44	7.60	10.66	10.63	10.74	11.67	12.24	11.28
	(6.03)	(5.76)	(6.04)	(4.52)	(4.39)	(4.56)	(5.09)	(4.85)	(5.25)	(6.02)	(5.62)	(6.41)
**Stearic acid 18:0**	3.96	3.92	3.99	2.89	2.82	2.90	4.14	4.08	4.19	4.33	4.46	4.14
	(2.26)	(2.33)	(2.28)	(2.04)	(1.81)	(2.12)	(2.01)	(1.87)	(1.96)	(2.42)	(2.45)	(2.43)
**Palmitoleic acid 16:1**	1.01	1.03	0.98	0.82	0.82	0.82	1.03	1.02	1.03	1.07	1.12	1.01
**n-7**	(0.58)	(0.63)	(0.54)	(0.49)	(0.51)	(0.49)	(0.47)	(0.54)	(0.52)	(0.63)	(0.68)	(0.57)
**Oleic acid 18:1 n-9**	21.73	22.23	21.46	16.55	16.84	16.28	22.29	22.51	21.47	23.83	24.57	22.83
	(10.98)	(10.77)	(11.27)	(11.10)	(10.89)	(12.03)	(9.81)	(9.91)	(9.77)	(10.97)	(10.71)	(10.95)
**Linoleic acid 18:2 n-6**	5.85	5.95	5.72	4.45	4.70	4.14	5.85	5.86	5.70	6.68	6.78	6.50
	(3.94)	(3.75)	(4.03)	(3.44)	(3.56)	(3.15)	(3.77)	(3.69)	(3.80)	(3.99)	(3.78)	(4.25)
**α-Linolenic acid 18:3**	0.44	0.45	0.42	0.35	0.36	0.34	0.43	0.44	0.42	0.49	0.49	0.47
**n-3**	(0.21)	(0.21)	(0.21)	(0.20)	(0.21)	(0.19)	(0.16)	(0.19)	(0.15)	(0.23)	(0.22)	(0.24)
**Arachidonic acid 20:4**	0.06	0.07	0.06	0.05	0.05	0.05	0.07	0.07	0.07	0.07	0.07	0.07
**n-6**	(0.06)	(0.07)	(0.06)	(0.05)	(0.06)	(0.05)	(0.07)	(0.07)	(0.07)	(0.06)	(0.07)	(0.06)
**Eicosapentaenoic**	0.01	0.01	0.01	0.01	0.01	0.01	0.01	0.01	0.01	0.01	0.01	0.02
**acid 20:5 n-3**	(0.10)	(0.10)	(0.10)	(0.10)	(0.11)	(0.08)	(0.10)	(0.09)	(0.10)	(0.10)	(0.10)	(0.10)
**Docosapentaenoic**	0.03	0.04	0.03	0.02	0.03	0.02	0.04	0.03	0.04	0.04	0.04	0.04
**acid 22:5 n-3**	(0.04)	(0.04)	(0.03)	(0.03)	(0.04)	(0.03)	(0.04)	(0.04)	(0.04)	(0.04)	(0.06)	(0.04)
**Docosahexaenoic**	0.02	0.02	0.03	0.02	0.04	0.01	0.02	0.01	0.03	0.02	0.02	0.04
**acid 22:6 n-3**	(0.14)	(0.14)	(0.14)	(0.14)	(0.15)	(0.12)	(0.14)	(0.13)	(0.14)	(0.15)	(0.15)	(0.15)
**Adapted Milk Consumers Cohort (AMS)**												
	**Total**			**1 to <3 years**			**3 to <6 years**			**6 to <10 years**		
	**Total**	**Boys**	**Girls**	**Total**	**Boys**	**Girls**	**Total**	**Boys**	**Girls**	**Total**	**Boys**	**Girls**
**Fatty acids (g)** ***n***	741	371	370	294	144	150	262	128	134	185	99	86
**Myristic acid 14:0**	1.02*	1.02*	1.03*	0.70	0.70*	0.70*	1.12	1.11*	1.12*	1.29	1.25*	1.30*

	(0.98)	(1.01)	(0.94)	(0.76)	(0.77)	(0.76)	(0.93)	(0.92)	(0.99)	(1.12)	(1.19)	(1.07)
**Palmitic acid 16:0**	7.85*	7.79*	8.04*	5.52	5.20*	5.62*	8.99	8.74*	9.35*	9.64	9.37*	9.83
	(5.28)	(5.22)	(5.31)	(4.32)	(4.64)	(4.25)	(4.99)	(4.81)	(5.22)	(5.19)	(5.42)	(5.07)
**Stearic acid 18:0**	2.95	2.91*	3.00*	2.00	1.99*	2.01*	3.52	3.26*	3.64*	3.76	3.72*	3.78
	(2.27)	(2.13)	(2.37)	(1.27)	(1.80)	(1.62)	(2.28)	(2.26)	(2.22)	(2.05)	(2.29)	(1.98)
**Palmitoleic acid 16:1**	0.76	0.77*	0.76*	0.61	0.58*	0.62*	0.84	0.84*	0.86*	0.88	0.86	0.90*
**n-7**	(0.52)	(0.54)	(0.51)	(0.46)	(0.48)	(0.45)	(0.51)	(0.49)	(0.56)	(0.47)	(0.51)	(0.42)
**Oleic acid 18:1 n-9**	17.74*	17.74*	17.75*	13.67	13.58*	13.92*	19.62	19.10*	20.83	22.64	22.76*	22.46
	(11.15)	(11.12)	(11.19)	(9.23)	(9.68)	(8.63)	(10.29)	(10.65)	(10.19)	(10.77)	(11.47)	(9.75)
**Linoleic acid 18:2 n-6**	5.15*	5.18*	5.12*	4.15	4.09	4.17	5.56	5.57	5.56	6.42	6.42	6.43
	(3.82)	(3.88)	(3.75)	(2.75)	(2.83)	(2.70)	(4.07)	(3.75)	(4.24)	(3.96)	(4.44)	(3.57)
**α-Linolenic acid 18:3**	0.42*	0.42	0.41	0.38	0.39	0.37	0.42	0.40	0.42	0.45	0.44*	0.46
**n-3**	(0.20)	(0.22)	(0.19)	(0.20)	(0.22)	(0.18)	(0.23)	(0.20)	(0.25)	(0.19)	(0.18)	(0.20)
**Arachidonic acid 20:4**	0.05*	0.05*	0.05*	0.04	0.04*	0.05	0.05	0.05*	0.06	0.06	0.06	0.06
**n-6**	(0.07)	(0.06)	(0.07)	(0.07)	(0.06)	(0.08)	(0.07)	(0.07)	(0.08)	(0.07)	(0.07)	(0.06)
**Eicosapentaenoic**	0.06*	0.05*	0.07*	0.01	0.01*	0.01	0.07	0.06*	0.08*	0.11	0.10*	0.11*
**acid 20:5 n-3**	(0.13)	(0.13)	(0.13)	(0.09)	(0.08)	(0.10)	(0.14)	(0.14)	(0.13)	(0.13)	(0.15)	(0.13)
**Docosapentaenoic**	0.03*	0.03*	0.03	0.02	0.02	0.03	0.03	0.03	0.03	0.04	0.04	0.04
**acid 22:5 n-3**	(0.04)	(0.04)	(0.04)	(0.03)	(0.03)	(0.03)	(0.04)	(0.05)	(0.04)	(0.04)	(0.04)	(0.04)
**Docosahexaenoic**	0.09*	0.08*	0.10*	0.10	0.09*	0.11*	0.09	0.08*	0.10*	0.08	0.08*	0.09*
**acid 22:6 n-3**	(0.14)	(0.15)	(0.15)	(0.14)	(0.14)	(0.16)	(0.15)	(0.15)	(0.14)	(0.12)	(0.15)	(0.11)

Average intake values for two 24 h dietary recalls were used. Results are expressed as median ± interquartile range (in brackets). Mann–Whitney U test was used to evaluate differences by total and sex groups between SRS and AMS) (significant differences are marked with an asterisk [*] in the median values of the AMS cohort). *p*-value < 0.05 was considered statistically significant.

Supplementary Table 4. Percentages of children meeting and not meeting European Food Safe Authority (EFSA) recommendations for main fatty acids by cohort and age group among plausible reporters of the Nutritional Study in Spanish Pediatric Population (EsNuPI) (*n* =1216) ¥.

Spanish Reference Cohort (SRS)

	**Total**				**1 to <3 years**				**3 to <6 years**				**6 to <10 years**				
	**n**	**% BR**	**% MR**	**% AR£**	**n**	**% BR**	**% MR**	**% AR£**	**n**	**% BR**	**% MR**	**% AR£**	**n**	**% BR**	**% MR**	**% AR£**	***p***
**Fat**	598	11.4	43.1	45.5	120	36.7^{a}	39.2^{ab}	24.2^{a}	211	11.4^{b}	51.2^{b}	37.4^{b}	267	0.0^{c}	38.6^{a}	61.4^{c}	<0.001
**Linoleic acid**	598	56.4	27.1	16.6	120	62.5	30.0	7.5^{a}	211	55.5	27.5	17.1^{b}	267	54.3	25.5	20.2^{b}	0.043
**α-Linolenic acid**	598	99.0	1.0	0.0	120	98.3	1.7	0.0	211	99.5	0.5	0.0	267	98.9	1.1	0.0	0.558
**EPA+DHA**	598	74.1	2.5	23.4	120	72.5	2.5	25.0	211	74.9	1.9	23.2	267	74.2	3.0	22.8	0.939

Adapted Milk Consumers Cohort (AMS)

	**Total**				**1 to <3 years**				**3 to <6 years**				**6 to <10 years**				
	**n**	**% BR**	**% MR**	**% AR£**	**n**	**% BR**	**% MR**	**% AR£**	**n**	**% BR**	**% MR**	**% AR£**	**n**	**% BR**	**% MR**	**% AR£**	***p***
**Total Fat**	618	26.7*	36.7*	36.6*	236	55.9^{a}*	35.6^{ab}	8.5^{a}*	224	14.7^{b}	42.9^{b}	42.4^{b}	158	0.0^{c}	29.7^{a}	70.3^{c}	<0.001
**Linoleic acid**	618	64.1*	26.1*	9.9*	236	75.8^{a}*	14.8^{a}*	9.3	224	51.8^{b}	35.3^{b}	12.9	158	63.9^{b}	29.7^{b}	6.3*	<0.001
**α-Linolenic acid**	618	97.1*	2.1*	0.8*	236	92.8^{a}	5.1^{a}	2.1	224	100.0^{b}	0.0^{b}	0.0	158	99.4^{b}	0.6^{b}	0.0	<0.001
**EPA+DHA**	618	59.1*	2.4*	38.5*	236	56.8*	3.8	39.4*	224	63.4*	1.3	35.3*	158	56.3*	1.9	41.8*	0.252

Percentage for inadequacy was calculated by comparing with EFSA recommendations. First column: percentage below recommendations (BR); second column: percentage meeting recommendations MR); third column: percentage above recommendations (AR). DHA, docosahexaenoic acid, 22:6 n-3; EPA, eicosapentaenoic acid, 20:5 n-3; NS, no significance. Results are expressed in percentage (%).¥ Individual usual intake for two 24 h dietary recalls was used for total fats, linoleic acid, α-linolenic acid, SFAs, and PUFAs. Average gram intake values from two 24 h dietary recalls were used for EPA+DHA. Chi-square test was used to evaluate differences by total and age group between SRS and AMS (significant differences are marked with an asterisk [*] in the percentage values of the AMS cohort). Chi-square test analysis was used to calculate differences among age groups within cohorts (significant differences are marked with superscript letters in the value of each age group that are meeting the recommendations). £ The percentage of children above the recommendations do not exceed the tolerable upper intake limit. *p-values* for this test are included in the last column of the table. *p*-value <0.05 was considered statistically significant.

Supplementary Table 5. Percentages of children meeting and not meeting Food and Agriculture Organization (FAO) recommendations for main fatty acids by cohort and age group among plausible reporters of the Nutritional Study in Spanish Pediatric Population (EsNuPI) (*n* = 1216‡) ¥.

	Spanish Reference Cohort (SRS)																
	Total				1 to <3 years				3 to <6 years				6 to <10 years				
	n	% BR	% MR	% AR£	n	% BR	% MR	% AR£	n	% BR	% MR	% AR£	n	% BR	% MR	% AR£	*p*
**Total Fat**	598	2.8	38.6	58.5	120	12.5[a]	30.0[a]	57.5	211	0.9[b]	43.6[b]	55.5	267	0.0[b]	38.6[ab]	61.4	<0.001
**SFAs ‡**	541	0.7	1.1	98.2	63	3.2	3.2	93.7	211	0.5	0.9	98.6	267	0.4	0.7	98.9	0.070
**PUFAs**	598	90.3	9.7	0.0	120	52.5[a]	47.5[a]	0.0	211	100.0[b]	0.0[b]	0.0	267	99.6[b]	0.4[b]	0.0	<0.001
**EPA+DHA**	598	68.6	5.2	26.3	120	64.2	5.8	30.0	211	67.3	4.3	28.4	267	71.5	5.6	22.8	0.482
	**Adapted Milk Consumers (AMS)**																
	Total				1 to <3 years				3 to <6 years				6 to <10 years				
	n	% BR	% MR	% AR£	n	% BR	% MR	% AR£	n	% BR	% MR	% AR£	n	% BR	% MR	% AR£	*p*
**Total Fat**	618	11.2*	34.1*	54.7*	236	29.2[a]*	33.1	37.7[a]*	224	0.0[b]	38.4	61.6[b]	158	0.0[b]	29.7	70.3[b]	<0.001
**SFAs ‡**	493	0.0*	2.8*	97.2*	111	0.0	7.2[a]	92.8[a]	224	0.0	1.8[b]	98.2[b]	158	0.0	1.3[b]	98.7[b]	0.007
**PUFAs**	618	79.8*	20.2*	0.0	236	47.0[a]	53.0[a]	0.0	224	100.0[b]	0.0[b]	0.0	158	100.0[b]	0.0[b]	0.0	<0.001
**EPA+DHA**	618	45.6*	12.1*	42.2*	236	49.6*	12.7*	37.7	224	40.6*	12.1*	47.3*	158	46.8*	11.4*	41.8*	0.314

Percentage for inadequacy was calculated by comparing with FAO recommendations. First column: percentage below recommendations (BR); second column: percentage meeting recommendations (MR); third column: percentage above recommendations (AR). DHA, docosahexaenoic acid 22:6 n-3; EPA, eicosapentaenoic acid 20:5 n-3; SFAs, saturated fatty acids; PUFAs: polyunsaturated fatty acids. Results are expressed in percentage (%).¥ Individual usual intake for two 24 h dietary recalls was used for total fats, linoleic acid, α-linolenic acid, SFAs, and PUFAs. Average gram intake values from two 24 h dietary recalls were used for EPA+DHA. Chi-square test was used to evaluate differences by total and age groups between SRS and AMS (significant differences are marked with an asterisk [*] in the median values of the AMS cohort). Chi-square test analysis was used to calculate differences among age groups within cohorts (significant differences are marked with superscript in median values of each age group). p-values for this test are included in the last column. *p*-value < 0.05 was considered statistically significant. **£** The percentage of children above the recommendations do not exceed the tolerable upper intake limit. ‡ Recommendations for SFAs intake available for 2 to <10 years, therefore, children <2 years were excluded (*n*= 1034).

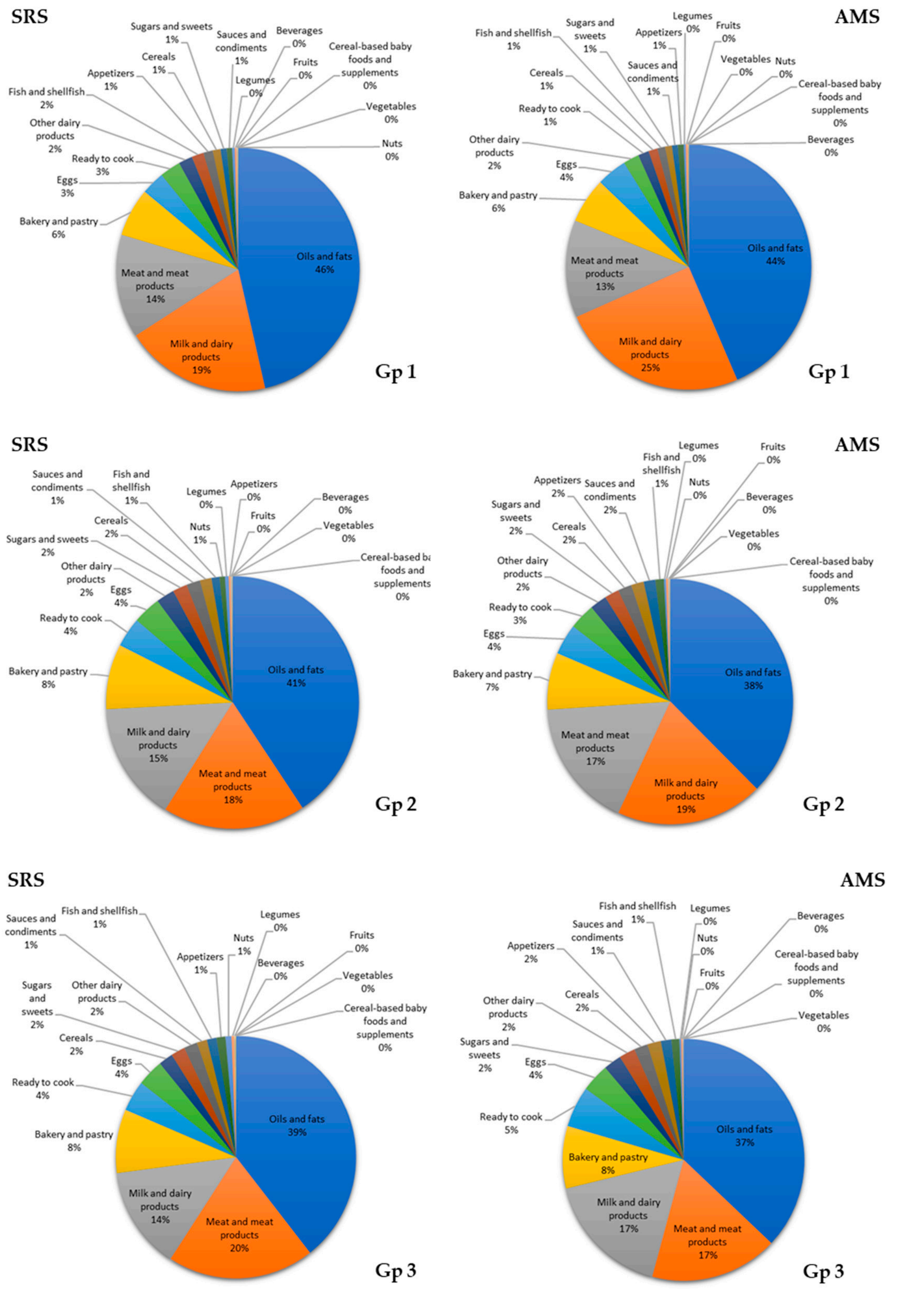

Supplementary Figure 1. Contribution (%) of the 18 food groups to monounsaturated fatty acids (MUFAs) intake in the EsNuPI study population (Spanish Reference Cohort (SRS) and Adapted Milk Consumers Cohort (AMS)) according to age group (Gp 1, 1 to <3 years; Gp 2, 3 to <6 years; Gp 3, 6 to <10 years).

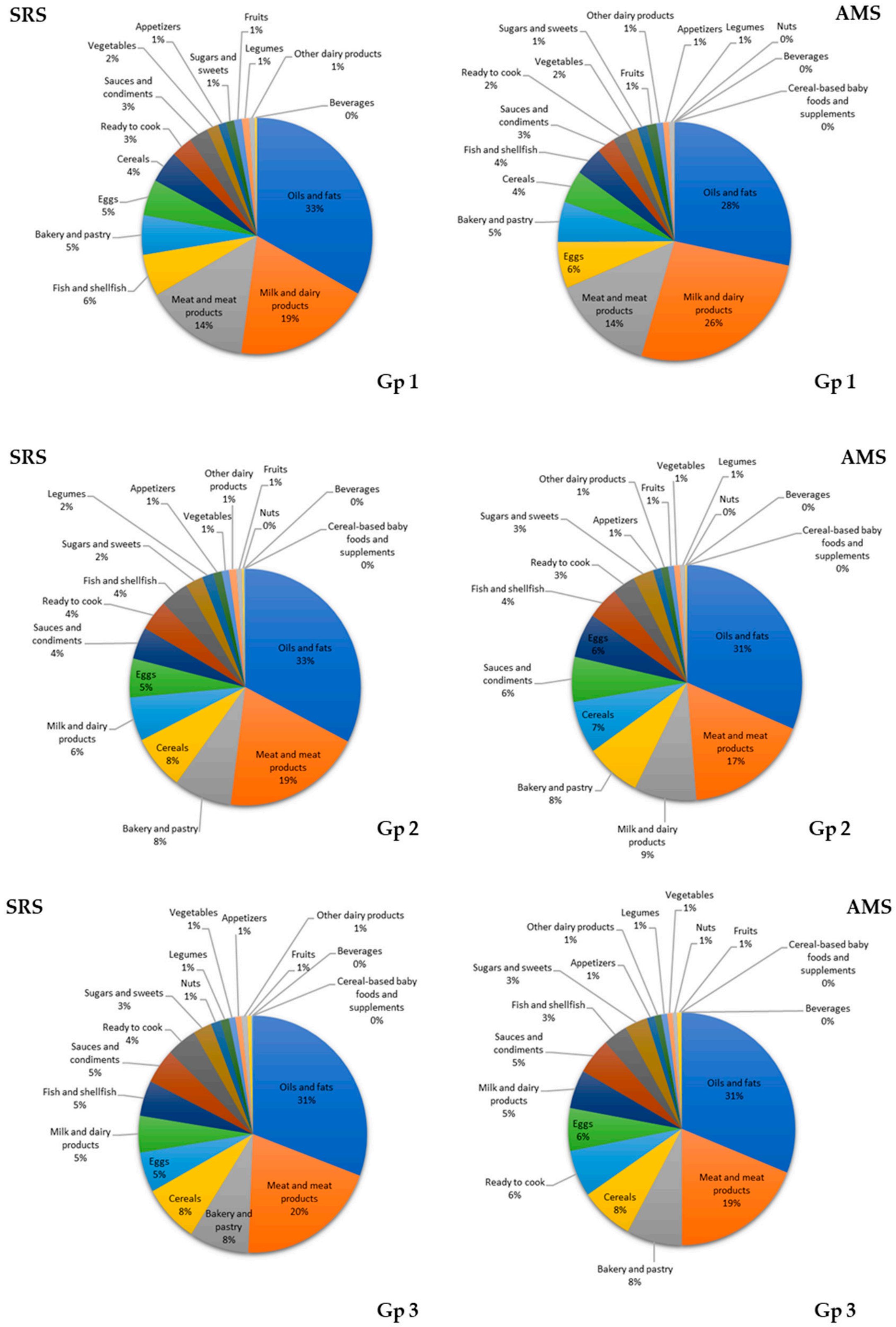

Supplementary Figure 2. Contribution (%) of the 18 food groups to polyunsaturated fatty acids (PUFAs) intake in the EsNuPI study population (Spanish Reference Cohort (SRS) and Adapted Milk Consumers Cohort (AMS)) according to age group (Gp 1, 1 to <3 years; Gp 2, 3 to <6 years; Gp 3, 6 to <10 years).

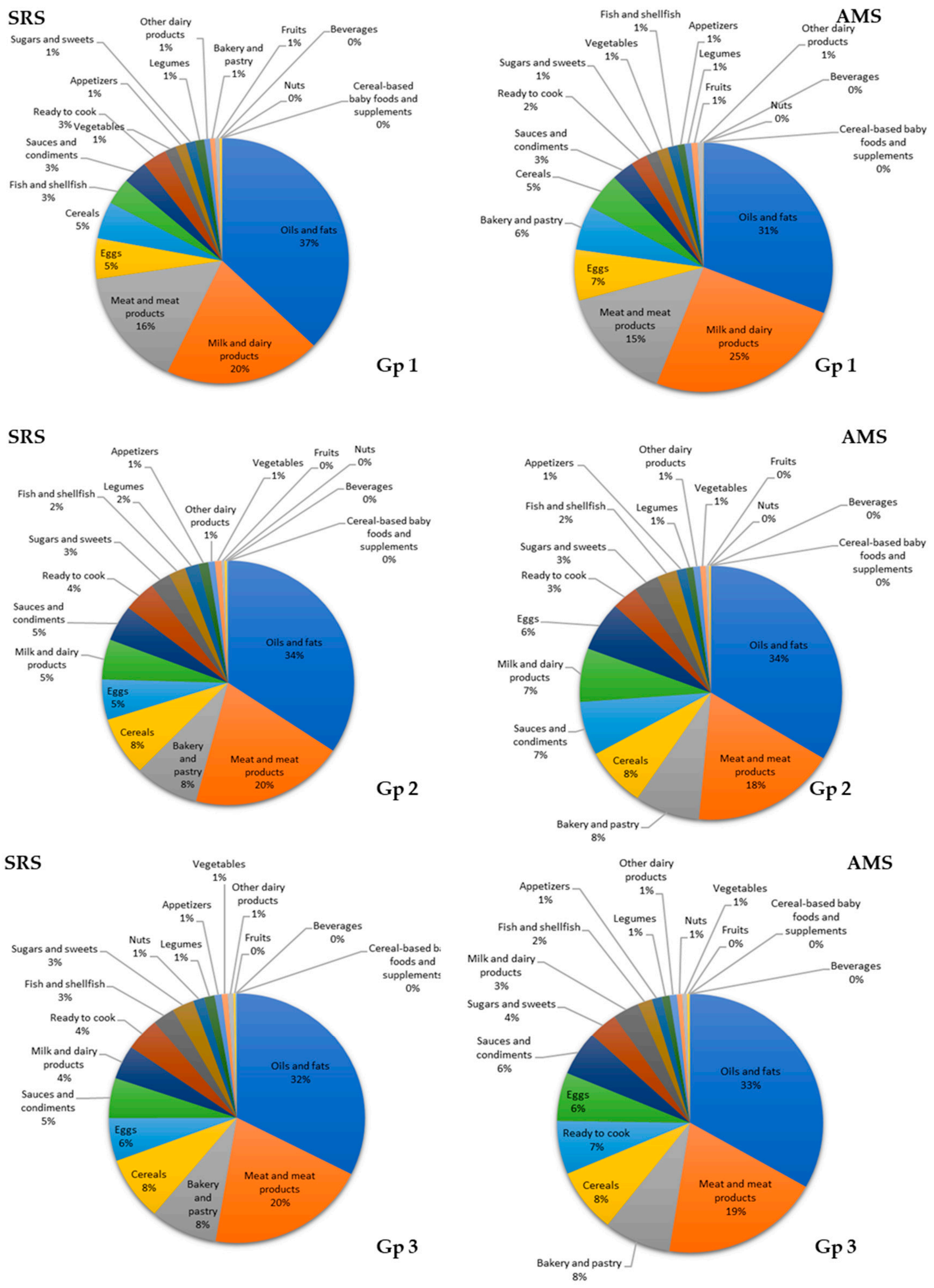

Supplementary Figure 3. Contribution (%) of the 18 food groups to n-6 intake in the EsNuPI study population (Spanish Reference Cohort (SRS) and Adapted Milk Consumers Cohort (AMS)) according to age group (Gp 1, 1 to <3 years; Gp 2, 3 to <6 years; Gp 3, 6 to <10 years).

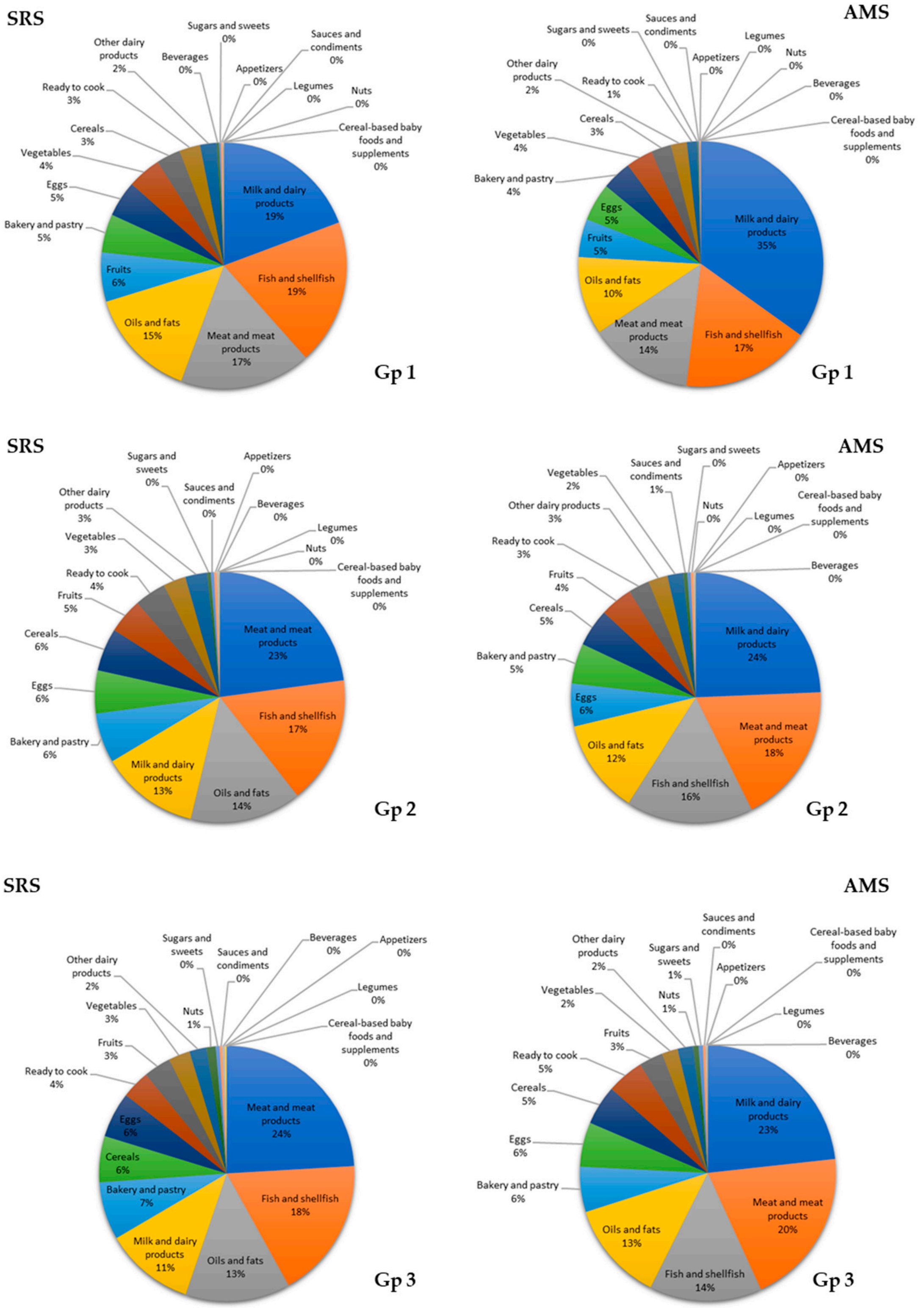

Supplementary Figure 4. Contribution (%) of the 18 food groups to n-3 intake in the EsNuPI study population (Spanish Reference Cohort (SRS) and Adapted Milk Consumers Cohort (AMS)) according to age group (Gp 1, 1 to <3 years; Gp 2, 3 to <6 years; Gp 3, 6 to <10 years).

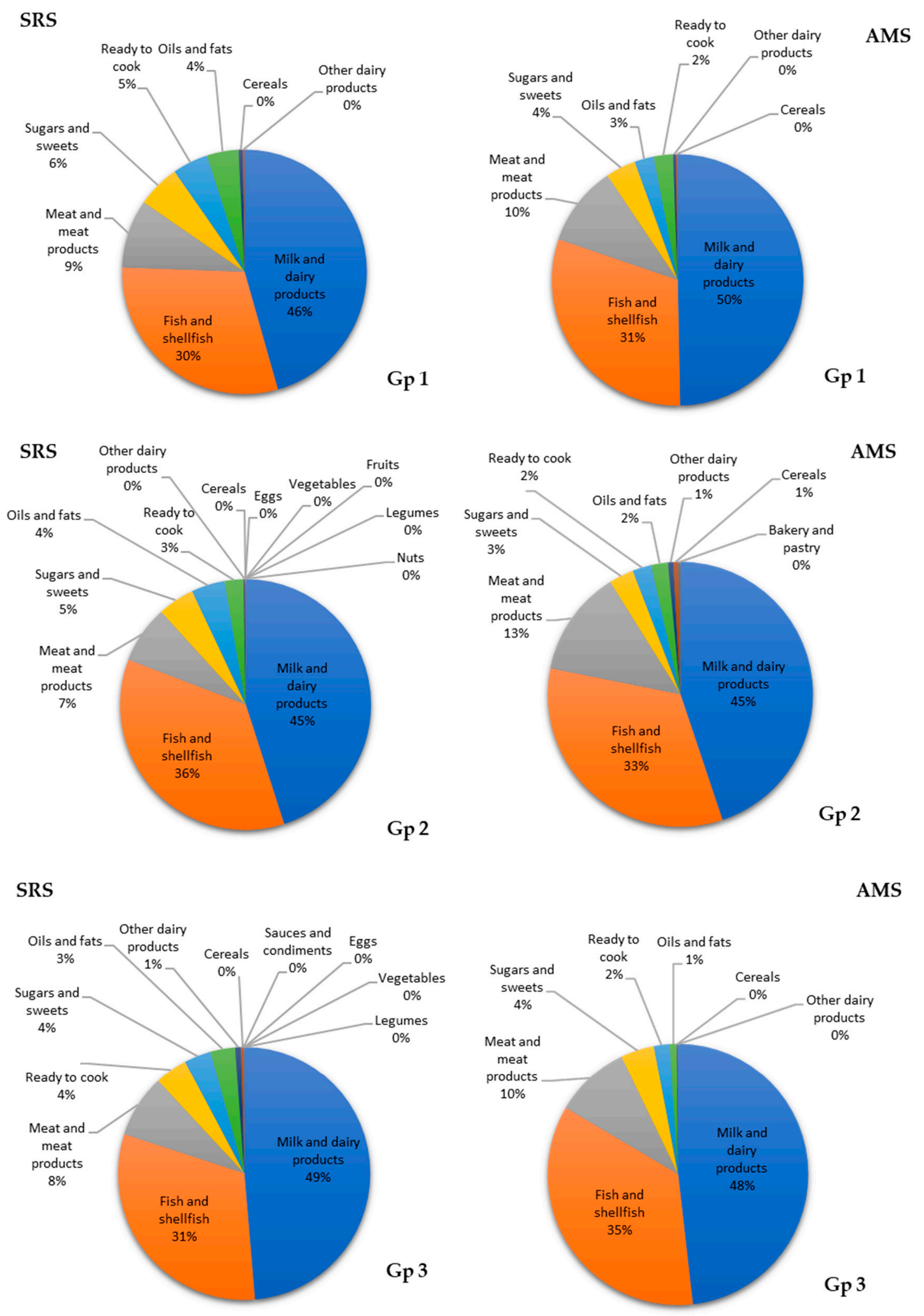

Supplementary Figure 5. Contribution (%) of the 18 food group to eicosapentaenoic acid (EPA) intake in the EsNuPI study population (Spanish Reference Cohort (SRS) and Adapted Milk Consumers Cohort (AMS)) according to age group (Gp 1, 1 to <3 years; Gp 2, 3 to <6 years; Gp 3, 6 to <10 years.
